# Multicolor emission based on a N, N′—Disubstituted dihydrodibenzo [a, c] phenazine crown ether macrocycle

**DOI:** 10.3389/fchem.2022.1087610

**Published:** 2022-12-05

**Authors:** Chang-Shun Ma, Chengyuan Yu, Cai-Xin Zhao, Shang-Wu Zhou, Ruirui Gu

**Affiliations:** Key Laboratory for Advanced Materials, Joint International Research Laboratory of Precision Chemistry and Molecular Engineering, Feringa Nobel Prize Scientist Joint Research Center, Frontiers Science Center for Materiobiology and Dynamic Chemistry, School of Chemistry and Molecular Engineering, Institute of Fine Chemicals, East China University of Science and Technology, Shanghai, China

**Keywords:** vibration-induced emission, host-guest interactions, multicolor emission, conformational adaptivity, supramolecular chemistry

## Abstract

Dynamic fluorophore 9,14-diphenyl-9,14-dihydrodibenzo[a,c]phenazine (DPAC) affords a new platform to produce diverse emission outputs. In this paper, a novel DPAC-containing crown ether macrocycle **D-6** is synthesized and characterized. Host-guest interactions of **D-6** with different ammonium guests produced a variety of fluorescence with hypsochromic shifts up to 130 nm, which are found to be affected by choice of solvent or guest and host/guest stoichiometry. Formation of supramolecular complexes were confirmed by UV-vis titration, ^1^H NMR and HRMS spectroscopy.

## Introduction

Supramolecular chemistry (Lehn, 2005; Stoddart, 2012; Yan et al., 2012; Yeung and Yam, 2015; Kolesnichenko and Anslyn, 2017; Liu et al., 2017; Zhou et al., 2017; Gu et al., 2018; Jana et al., 2018; Xia et al., 2020; Gu and Lehn, 2021; Shen et al., 2021; Zhang et al., 2021; Zhang et al., 2022a; Zhang et al., 2022b; Huang et al., 2022) is undergoing tremendous speed of development, being important tools to modulate optical properties of chemical systems. Multicolor emission has been extensively investigated over the past decade due to its considerable application prospects in displays (Nie et al., 2022; Zou et al., 2022), illumination (Lee et al., 2016; Zhang et al., 2019a; Gong et al., 2019), molecular/ion recognition (Wang et al., 2012; Li et al., 2017; Li et al., 2018a; Zhang et al., 2019b; Chen et al., 2019; Sun et al., 2020), and biosensing (Zhou et al., 2019; Dong et al., 2020; Yan et al., 2021; Du and Wei, 2022). Doping (Nie et al., 2022) or hybridizing (Cui et al., 2017) of different fluorophores are effective methods to generate multicolor emission, these systems usually requires more than a single excitation wavelength or stimulation methods to achieve multicolor emissions. However, many chemical systems exhibiting multicolor emission have been constructed in the presence of only one chromophore by the modulation of host-guest interaction (Zhang et al., 2016; Li et al., 2018a; Wang et al., 2020a; Wang et al., 2020b; Sun et al., 2020; Wu et al., 2020; Zhang et al., 2022c; Yu et al., 2022), pH (Li et al., 2018b; Bai et al., 2019; Radunz et al., 2019; Liu et al., 2021), hydrogen bonding (Wu et al., 2019; Tao et al., 2020), metal coordination (Lee et al., 2017), and other methods (Feng et al., 2015; Huang et al., 2015; Shi et al., 2018; Wang et al., 2019; Naren et al., 2020; Guo et al., 2021; Wang et al., 2022a; Wang et al., 2022b; Qiu et al., 2022; Zong et al., 2022). Although progresses have been made in the study of single-chromophore multicolor emission, it is still of great value to develop new and controllable multicolor emission systems for a wider range of application scenarios.

N,N′—diphenyl—dihydrodibenzo [a,c] phenazines (DPAC) possesses unique photophysical properties including the double fluorescence emission, large stokes shift and remarkable responsiveness to various environmental stimuli (Zhang et al., 2015; Zhang et al., 2020). In solution, the unique saddle-shaped structures of DPAC units undergo dynamic light-induced planarization processes upon photoexcitation and emit orange-red fluorescence. When such vibrational motions of the molecules are restricted, e.g., in the solid state, only the intrinsic blue fluorescence could be detected. The described vibration-induced emission (VIE) behavior of the DPAC chromophore has provided a new platform for chemists to build multicolor emission systems by meticulous control of its molecular geometry (Huang et al., 2015; Shi et al., 2018; Zhang et al., 2018). For example, in an inspiring work of Tian and Chou (Chen et al., 2017), a number of DPAC-based macrocycles with various sizes were systematically investigated. The different degrees of constraint of the DPAC units resulted in various emissions from 490 nm to 625 nm, showing the great potential of these chemically locked DPAC containing macrocycles in both fundamental studies and optical applications.

**SCHEME 1:**
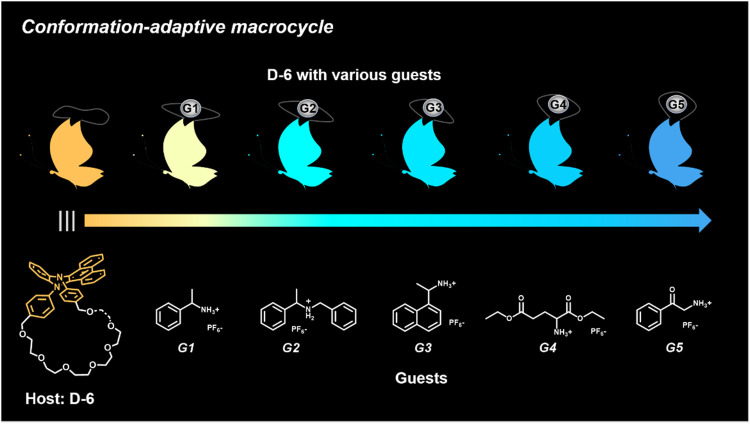
Chemical structures of conformation-adaptive macrocycle host **D-6** and ammonium guests **G1**-**G5**, and schematic representations of their combinations exhibiting diverse emission from orange to blue when equimolar **D-6** and different guests were mixed, respectively.

Herein, we designed and synthesized a large-size DPAC-based crown ether macrocycle D-6 whose dynamic DPAC chromophore was covalently locked by a conformational flexible hexaethylene glycol chain (see Scheme 1 for the structure of D-6). The electron-rich cavity of this crown ether was able to supramolecularly combine electron-deficient molecules/ions through host-guest interactions and subsequently increase the constrain of the DPAC wings. Relying on this understanding, we managed to produce multicolor fluorescent signals from orange to blue by: 1) respectively mixing the macrocycle with different ammonium guests G1-G5 (Scheme 1); 2) titration of an ammonium guest G5 to the emissive macrocycle D-6. White light emission was also obtained in this work in a specific stoichiometry of D-6 and G5.

## Experiment section

### Synthesis of DPAC-crown ether ring (D-6) and guests

The macrocycle D-6 was synthesized in three steps from N, N′- diphenyl dihydrodibenzo [a, c] phenazine (the synthetic route is shown in Supplementary Scheme S1). Compound 3 were prepared referring to the method described in the literature (Zhang et al., 2015). In the final step, compound D-6 was produced with a 40% yield by the Williamson etherification reaction of compound 3 and hexaethylene glycol di (p-toluenesulfonate) under the templation of sodium hydride. ^1^H NMR, ^13^C NMR, and high-resolution mass spectrometry (HRMS) were used to confirm the chemical structure of D-6 (Supplementary Figures S13–S15). The five ammonium hexafluorophosphates G1-G5 involved in this paper were obtained by protonation and ion exchange of commercially available amines, or direct ion exchange of commercially available ammonium hydrochloride salts, respectively (see Supplementary Material for experimental details).

## Materials and methods

The ^1^H NMR and ^13^C NMR data were measured by AV-400 NMR spectrometer made by Brucker Company, in which the internal standard reference was tetramethylsilane (TMS), and the detection temperature was room temperature (25°C, 298 K) unless otherwise specified. High resolution mass spectrometry (HRMS) was performed by Waters LCT Premier XE mass spectrometer, in which electrospray ionization (ESI) was used for ionization. The UV/Vis absorption spectra data were documented by a Shimadzu UV-2600 UV-Vis spectrophotometer and the fluorescent spectra were acquired by a Shimadzu RF6000 spectro fluorophotometer.

## Results and discussion

**FIGURE 1:**
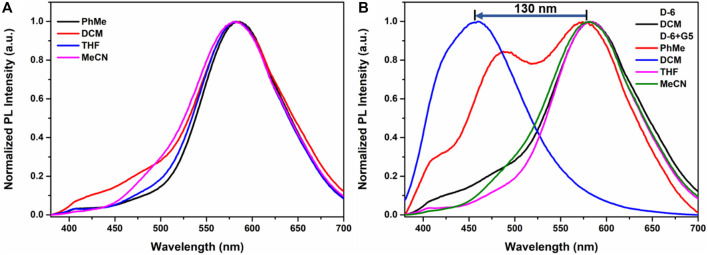
**(A)** Emission spectra of **D-6** in different solvents. **(B)** Emission spectra of the mixtures of 1 eq. **D-6** and 1 eq. **G5** in different solvents.

It should be noted that **D-6** is not the first DPAC-involving crown ether we investigate. In a previous work of Qu group (Yang et al., 2021), a smaller sized DPAC-ring with pentaethylene glycol backbone was inserted a dibenzylammonium guest to show the adaptive emission of the DPAC-ring (in contrast, the effect of dibenzylammonium salt on **D-6** is detailed in Supplementary Figure S12). There, the host-guest interaction only caused a small spectral shift of 13 nm (from 490 nm to 477 nm) in acetonitrile with a small visual variation from light blue to blue. In comparison, the emission of the present macrocycle **D-6** in acetonitrile reaches 584 nm (Figure 1A), 94 nm longer than the previously reported macrocycle, indicating a smaller constraint of **D-6** in the guest-free state. Different solvents including toluene, dichloromethane, tetrahydrofuran, and acetonitrile were tested here and no significant disparity was generated (Figure 1A). Surprisingly, when **D-6** in these solvents were respectively added **G5**, a drastic blue shift of 130 nm was detected only when dichloromethane was utilized as the solvent (Figure 1B), achieving a 10-fold dynamic variation in emission wavelength comparing to the previous work. Due to this huge variation which is beneficial to generate multicolor emissions, dichloromethane was chosen as the main solvent in the present work. And in every case, a volume fraction of 5% methanol was added to the solutions of ammonium guests in order to better dissolve the guests.

UV-vis spectroscopy and fluorescence spectroscopy were utilized to study the photophysical properties of **D-6**. All the spectra were recorded at room temperature. The maximum UV absorbance of **D-6** is approximately 352 nm which is attributed to the DPAC chromophore rather than the crown ether moiety (Supplementary Figure S1A), in line with the earlier studies on DPAC systems showing no apparent absorption data above 400 nm. Upon excitation of 360 nm UV light, the solution of D-6 emitted orange fluorescence at 584 nm as shown in Figure 1A, suggesting a weak constraint of DPAC wings.

The responsiveness of **D-6** to the supramolecular guests **G1**-**G5** were then studied. First, UV-vis titrations were carried out to investigate the supramolecular complexations and to determine the binding ratios of the host macrocycle and the guests (Supplementary Figures S2–S6). All the absorbances underwent gradual decreases when the guests were added to the solutions of **D-6**. Meanwhile, hypsochromic shifts of ∼5 nm could be observed in the cases of **G3**-**G5**, indicating stronger combinations of **D-6** with them. In all cases, when the molar ratios of guest cations and the host were 1:1, the Job’s Plot curves reached the maximum values, revealing that all the guests were hosted by **D-6** macrocycle in the ratio of 1:1. Meanwhile, the quantum yields and fluorescence lifetimes of D-6 and the host-guest complexes of different guests were also measured as detailed in Supplementary Table S1.

**FIGURE 2:**
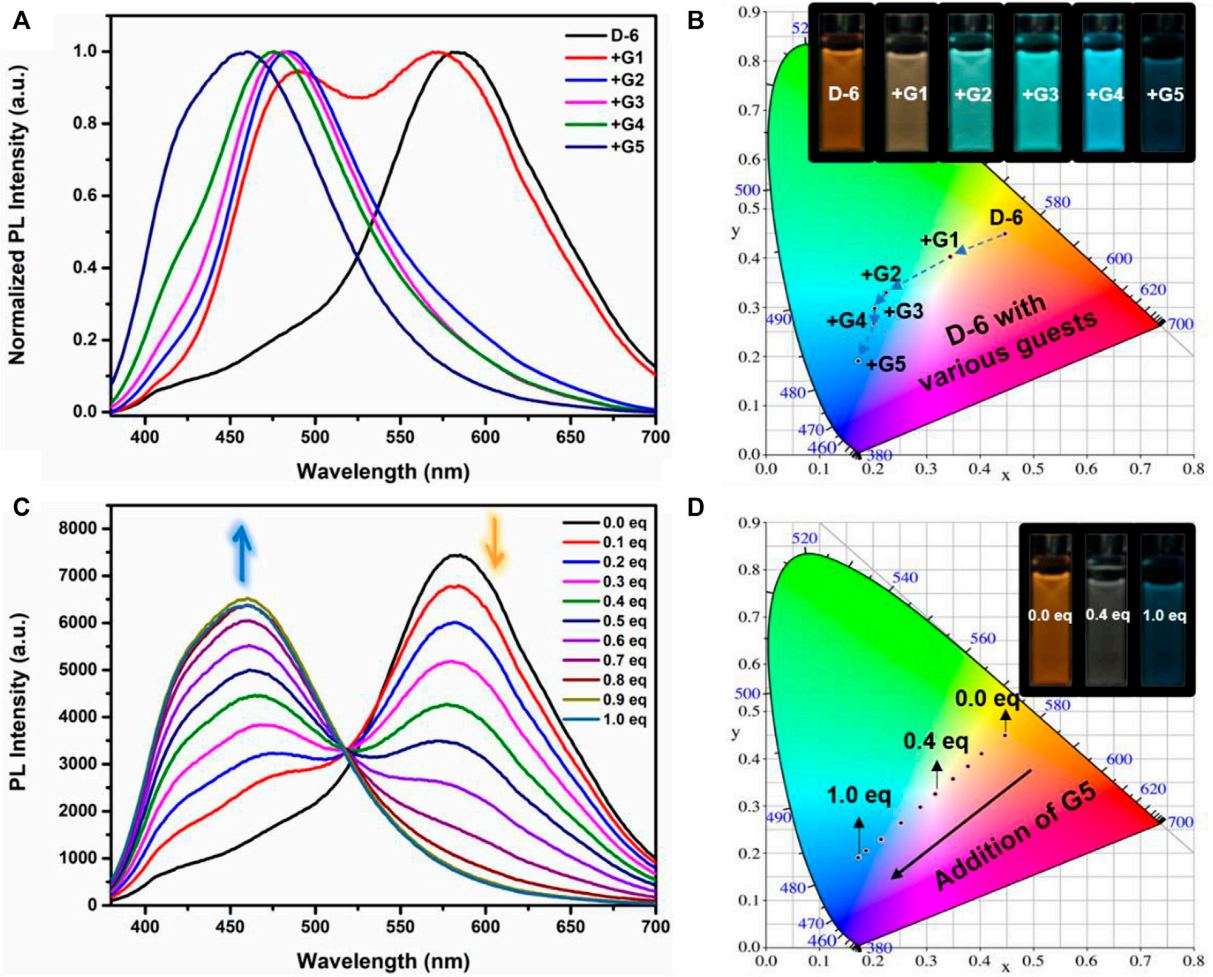
**(A)** Normalized fluorescence emission spectra of **D-6** alone (black) and in the presence of various guests [in dichloromethane; (**D-6**) = 10 μM; λ_ex_ = 360 nm]. **(B)** Chromaticity coordinates (CIE) of **D-6** and the host-guest complexes in dichloromethane. Inset: images of **D-6** and **D-6**⊃Guests upon irradiation with 360 nm UV light. **(C)** Fluorescence curves upon titration of 0.1 eq. **G5** to the solution of **D-6** [in dichloromethane; λ_ex_ = 360 nm; (**D-6**) = 10 μM]. **(D)** CIE diagram of **D-6** solutions containing various quantities of **G5** (every 0.1 eq.). Inset: images of solutions of **D-6** containing 0, 0.4, and 1.0 eq. **G5** under irradiation with 360 nm UV light.

Fluorescence spectra of the host-guest mixtures were then recorded at room temperature to examine the impact of supramolecular complexation on the fluorescence characteristics of **D-6** macrocycle. Different degrees of variations, both visually and spectrally, were observed when the solutions of **D-6** in dichloromethane were added equimolar ammonium salts respectively. The emission spectra of D-6 before and after the addition of guests were transformed into CIE coordinates: **D-6** (0.45, 0.45), **G1** (0.34, 0.40), **G2** (0.22, 0.33), **G3** (0.20, 0.30), **G4** (0.20, 0.27), and **G5** (0.17, 0.19) (Figure 2B and Supplementary Figure S12). The emission of **D-6** and **G1** was pale yellow with two peaks at 490 nm and 571 nm (red curve in Figure 2A), probably due to their insufficient host-guest complexation. In comparison, addition of all the other four guests **G2**-**G5** brought huge hypsochromic shifts in emission wavelength (100, 101, 106, and 130 nm for **G2**, **G3**, **G4**, and **G5**, respectively), demonstrating the large impact of host-guest interactions. In particular, the addition of **G5** to **D-6** produced the largest shift of 130 nm from 584 nm (orange) to 454 nm (dark blue). The large variations of emission color in response to different guests were most likely caused by the conformational adaptation of **D-6**. The originally relaxed ethylene glycol backbone underwent a stronger resistance in tension after the insertion of the guests. Simultaneously, the wings of the DPAC unit were constrained to perform light-induced structural planarization, resulting in the changes of fluorescence. Potentially, the **D-6** macrocycle could be used as a supramolecular fluorescent probe to distinguish different ammonium salts.

Fluorometric titration of **G5** to **D-6** was then carried out. As is clearly shown in Figure 2C, upon gradient addition of 0.1 eq. **G5**, the main emission peak of **D-6** at 584 nm gradually decreased while a peak around 470 nm arose and increased simultaneously. Eventually, the new peak stopped to increase when 1 eq. guest was added. Additionally, noticeable visual changes could be observed after each 0.1 equivalent **G5** was added. The fluorescence spectra discussed above were also translated to CIE coordinates as following: 0 eq. (0.44, 0.44), 0.1 eq. (0.40, 0.41), 0.2 eq. (0.38, 0.38), 0.3 eq. (0.35, 0.36), 0.4 eq. (0.32, 0.33), 0.5 eq. (0.29, 0.3), 0.6 eq. (0.25, 0.26), 0.7 eq. (0.21, 0.23), 0.8 eq. (0.17, 0.19), 0.9 eq. (0.17, 0.19), 1.0 eq. (0.17, 0.19). From the CIE chromaticity diagram (Figure 2D), a linear variation in color was accomplished, including the white light intermediate spot at (0.32, 0.33). Thus, multicolor emissions are efficiently obtained in this system by simple addition of the supramolecular guest to the host.

**FIGURE 3:**
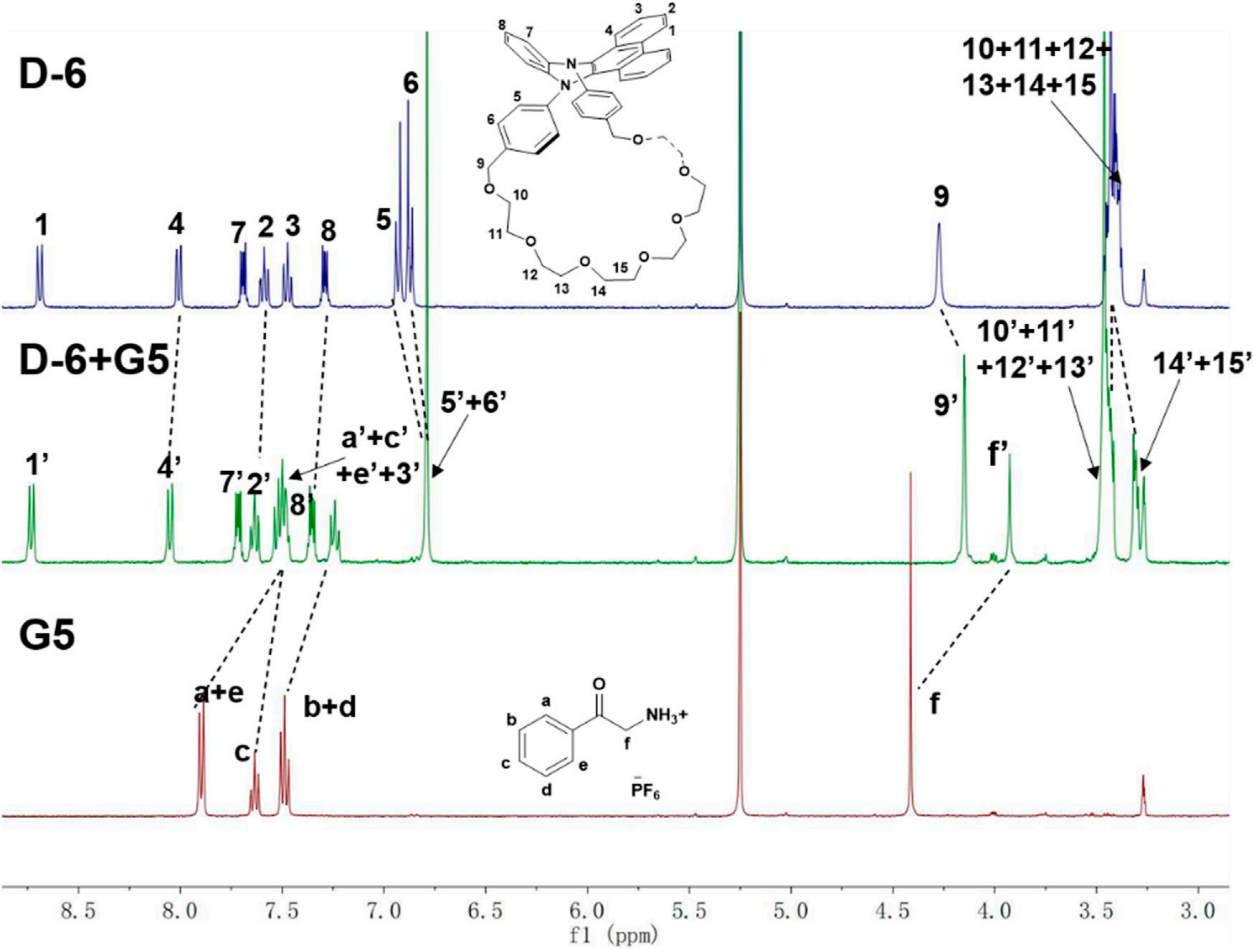
The 400 MHz ^1^H NMR of **D-6** (blue), **G5** (red) and their equimolar mixture **D-6** + **G5** (green) in dichloromethane-*d2/*methanol*-d4* (v/v = 95:5).

The formation of the host-guest complexes was further confirmed by 400 MHz ^1^H NMR (see Supplementary Figures S16–S22 for the NMR spectra of the mixtures of **D-6** and **G1**-**G4**, respectively). The spectra of **D-6** (blue), **G5** (red), and their equimolar mixture (green) were shown in Figure 3. After the mixing of **G5** and **D-6**, the methylene proton H_f_ of **G5** is observed to significantly upshifted by 0.48 ppm. Similarly, all the phenyl protons of **G5** shifted to the higher field (0.37 ppm for H_a,e_, 0.24 ppm for H_b,d_, and 0.13 ppm for H_c_). Meanwhile, all of the protons of **D-6** underwent displacements, especially H_5,6_, H_9_, and H_14,15_ (−0.11 ppm for H_5,6_, −0.12 ppm for H_9_, and ∼−0.09 ppm for H_14,15_). All the changes could be explained by the insertion of **G5** into the cavity of **D-6** and the consequent formation of the host-guest structure. Similar spectral variations were also found in the other four cases. In addition, the molecular ion peaks found for **D-6** and its visitors in the HRMS data also support the complexation of them (Supplementary Figures S17–S25).

By processing the spectra of UV-vis titrations (respective addition of **G1**-**G5** into the solutions of **D-6**) using nonlinear regression methods (Thordarson, 2011), a number of corresponding binding constants were obtained (Table 1). The large magnitudes of binding constants further clarify the formation of supramolecular complexes. It is already known from the fluorescence spectra of **D-6** along and with the five guests that the degrees of hypsochromic shifts increase in the order of **G1**-**G5**. Interestingly, the binding constants are perfectly in line with this sequence, showing the binding constant dependance of color change. The differences of binding constants were considered to be partially due to the different electron deficiency of the ammoniums. Comparing to **G1**-**G3**, the carbonyl groups of **G4** and **G5** increase the electron deficiency of their ammonium sites and consequently bring higher affinity in the complexation with the electron rich crown ether cavity. However, the relationship of the chemical inputs and the emission colors in the present work can hardly be contributed to only this reason. The long hexaethylene glycol backbone gives access to the guests with different sizes while the strong topological flexibility of the backbone gives possibility to the conformational adaptation of the DPAC unit and the whole macrocycle. Relationally, the different rigidities of the guests could also affect the binding geometry and the binding constants.

**TABLE 1:** The binding constants of the guest cations and the fluorescent macrocycle **D-6**.

Guest		Binding constants
**G1**	, 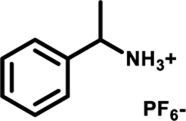	(4.11 ± 0.57)*10^6^
**G2**	, 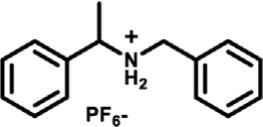	(4.78 ± 0.52)*10^6^
**G3**	, 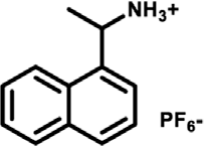	(2.20 ± 0.97)*10^7^
**G4**	, 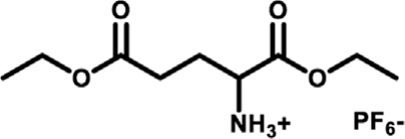	(4.68 ± 2.29)*10^7^
**G5**	, 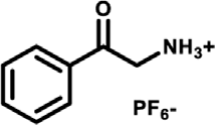	(7.09 ± 3.40)*10^7^

## Conclusion

In conclusion, relying on the light induced structural planarization of DPAC derivatives, we synthesized a new emissive macrocycle **D-6** as a conformation-adaptive supramolecular host. By the respective incorporation of various ammonium guests, multicolor emission from orange to white to deep blue was accomplished. Solvent and host/guest stoichiometry were found to be effectors that influence the optical outputs. The supramolecular host-guest complexation was confirmed by UV-vis titration, ^1^H NMR, and HRMS data. The guest-dependent emission of **D-6** shown in this work is potential to distinguish different ammoniums, which would be continuously studied by our group in future. The use of supramolecular chemistry to modulate emission wavelengths over a broad range afford an effective way to obtain multicolor emission within a less complicated system.

## Data Availability

The original contributions presented in the study are included in the article/Supplementary Material, further inquiries can be directed to the corresponding author.
