# Supplementary material for: Multicolor emission based on a N, N′—Disubstituted dihydrodibenzo [a, c] phenazine crown ether macrocycle
Source: Front Chem. 2022 Dec 5;10:1087610. doi: 10.3389/fchem.2022.1087610 (PMC9760862; doi:10.3389/fchem.2022.1087610)
Supplement: Supplementary file 1 [file Presentation1.PDF]

Supporting Information for

Multicolor Emission Based on a N, N' - Disubstituted Dihydrodibenzo [a, c] Phenazine Crown Ether Macrocycle

Chang-Shun Ma, Chengyuan Yu, Cai-Xin Zhao, Shang-Wu Zhou, Ruirui Gu*.

Key Laboratory for Advanced Materials and Joint International Research Laboratory of Precision Chemistry and Molecular Engineering, Feringa Nobel Prize Scientist Joint Research Center, Frontiers Science Center for Materiobiology and Dynamic Chemistry, Institute of Fine Chemicals, School of Chemistry and Molecular Engineering, East China University of Science and Technology, Shanghai, China.

Contents

1.Materials and Methods 3
2. Synthesis of D-6 3
3. Synthesis of G1 4
4. Synthesis of G2 4
5. Synthesis of G3 4
6. Synthesis of G4 4
7. Synthesis of G5 5
8. UV/Vis and Fluorescence Spectra of D-6 5
9. Job's Plot curves of D-6 and various ammonium salts 5
10. UV/Vis titration spectra of various ammonium salts for D-6. Determination of the association constant (Ka) between D-6 and Guests. http://app.supramolecular.org/bindfit/. 7
11. Optical properties and fluorescence titration spectra of various ammonium salts for D-6 12
12. ^{1}H NMR, ^{13}C NMR, and HRMS spectra 15

1.Materials and Methods

Materials. All chemicals were used as received from Adamas-beta, Acros, Aldrich, or Merck. All solvents were reagent grade, which were dried and distilled prior to use according to standard procedures. The thin layer chromatography (TLCs) were performed on Greagent plates (MFCD00011232, 0.2 mm) to monitor the whole processes. Flash column chromatography was performed using silica gel (Greagent, 200-300 mesh) to purified crude products. **Compound 3** was prepared using literature procedures.

Instruments. The molecular structures of the unknown compounds were confirmed via NMR spectroscopies and High Resolution ESI mass spectroscopy. ^{1}H NMR spectra and ^{13}C NMR were recorded on a Brücker AM400. The (HR-ESI) mass spectra were tested on a LCT Premier XE mass spectrometer. The UV/Vis absorption spectra data were documented by a Shimadzu UV-2600 UV-Vis spectrophotometer and the fluorescent spectra were acquired by a Shimadzu RF6000 spectro fluorophotometer.

Binding Constants: The binding constants were calculated using the method reported on website http://app.supramolecular.org/bindfit/

2. Synthesis of D-6

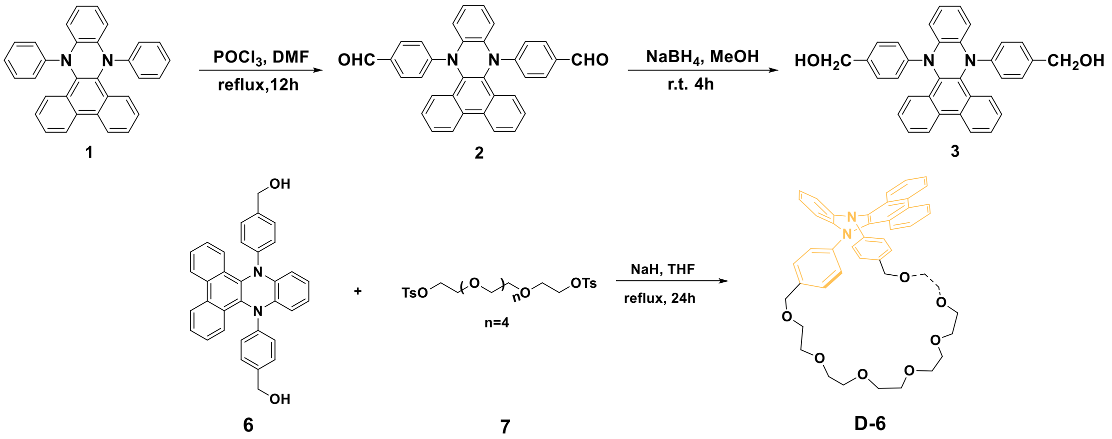

To the solution of 3 (500 mg, 1.0 mmol) was added 60% NaH (160 mg, 4.0 mmol) in anhydrous THF (10 mL). The reaction mixture was stirred at 60 °C for 2 h under Ar atmosphere. Hexaethylene glycol di(p-toluenesulfonate) (695 mg, 1.5 mmol) was added to the solution of mixture , and the reaction mixture was continuously stirred at 70 °C overnight. After cooling down to room temperature, the mixture was added ice water (1 mL) and organic solvent was evaporated under reduced pressure. The water phase was extracted with DCM (2×20 mL) and the combined organic layer was washed by brine (2×20 mL), then dried with anhydrous Na_2SO_4, purified by column chromatography (SiO_2, dichloromethane/methanol, 100:1, v/v) to give compound **D-6** as a yellow solid (299.5 mg, 40%). ^{1}H NMR (400 MHz, $CDCl_3$, 298 K) δ (ppm): 8.74 (d, J = 8.0 Hz, 2H), 8.11 (d, J = 7.2 Hz, 2H), 7.74 (dd, J_1= 3.2 Hz, J_2= 5.6 Hz, 2H), 7.65 (t, J = 7.2 Hz, 2H), 7.55 (t, J = 8.0 Hz, 2H), 7.35 (dd, J_1 = 3.6 Hz, J_2 = 6.0 Hz, 2H), 7.01 (d, J = 8.8 Hz, 4H), 6.92 (d, J = 8.8 Hz, 4H), 4.391 (s, 4H), 3.57-3.54 (m, 20H), 3.46-3.44 (m, 4H). ^{13}C NMR (100 MHz, $CDCl_3$, 298 K) δ (ppm): 147.3, 144.9, 138.2, 129.9, 129.4, 128.6, 127.4, 127.0, 126.6, 125.4, 124.6, 123.0, 116.6, 72.7, 70.7, 70.6, 69.0.

[M+Na]+ calcd for $C_{46}H_{48}N_2NaO_7^+$,763.3359; found, 763.3356.

3. Synthesis of G1

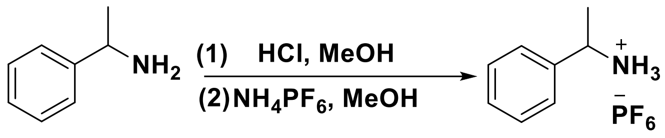

G1

In a 50 ml round-bottomed flask, Methylbenzylamine(1.0 g, 8 mmol) was added, followed by methanol (25 ml), hydrochloric acid(2ml, 20 mmol), and stirred at room temperature for 1 hour. The solvent was then extracted under vacuum, methanol (3 ml) was added, 10 ml of saturated ammonium hexafluorophosphonate solution was put into the mixture, and it was stirred for 1 hour. The solvent was removed in vacuo after the ethyl acetate extraction (3×10 ml), and the target component **G1**(2.09 g, 95%) was extracted using column chromatography. ^{1}H NMR (400 MHz, DMSO-d6, 298 K) (ppm): 8.04 (s, 2.7H), 7.35-7.50 (m, 5H), 1.47(d, 3H).

4. Synthesis of G2

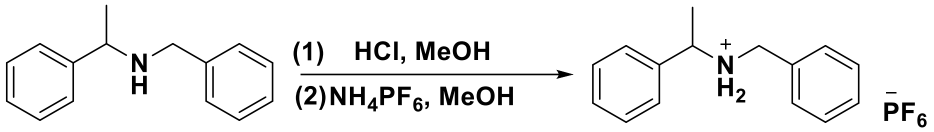

G2

The synthesis of **G2** is carried out according to the synthesis of **G1**. The synthesis of **G3** is carried out according to the synthesis of **G1**. ^{1}H NMR (400 MHz, DMSO-d6, 298 K) (ppm): 9.22 (s, 2.H), 7.34-7.57(m, 10H), 4.41(s, 1H), 4.11(d, 1H), 3.85(d, 2H), 1.59(d, 3H).

5. Synthesis of G3

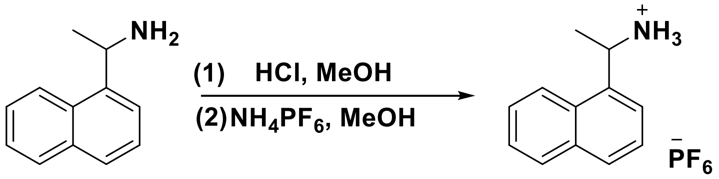

G3

The synthesis of **G3** is carried out according to the synthesis of **G1**. The synthesis of **G3** is carried out according to the synthesis of **G1**. ^{1}H NMR (400 MHz, DMSO-d6, 298 K) (ppm): 8.22 (s, 1.34H), 8.20 (q, 2H), 7.57-7.71(m, 4H), 5.29(q, 1H), 1.60(d, 3H).

6. Synthesis of G4

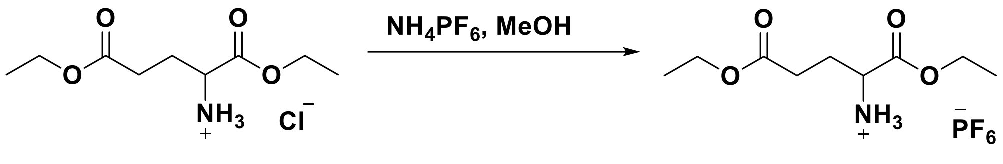

G4

The synthesis of **G4** is carried out according to the synthesis of **G1**. ^{1}H NMR (400 MHz, DMSO-d6, 298 K) (ppm): 8.03 (s, 2.42H), 4.20 (q, 2H), 4.07(q, 4H), 4.01(t, 1H), 2.39-2.57(m, 2H), 1.90-2.09(m, 2H), 1.15-1.28(m, 6H).

7. Synthesis of G5

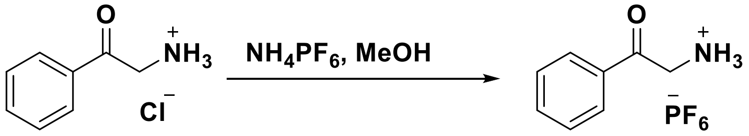

G5

The synthesis of **G5** is carried out according to the synthesis of **G1**. The synthesis of **G4** is carried out according to the synthesis of **G1**. ^{1}H NMR (400 MHz, DMSO-d6, 298 K) (ppm): 8.20 (s, 2.67H), 8.02 (d, 2H), 7.75(d, 2H), 7.61(t, 2H), 4.62(s, 2H).

8. UV/Vis and Fluorescence Spectra of D-6

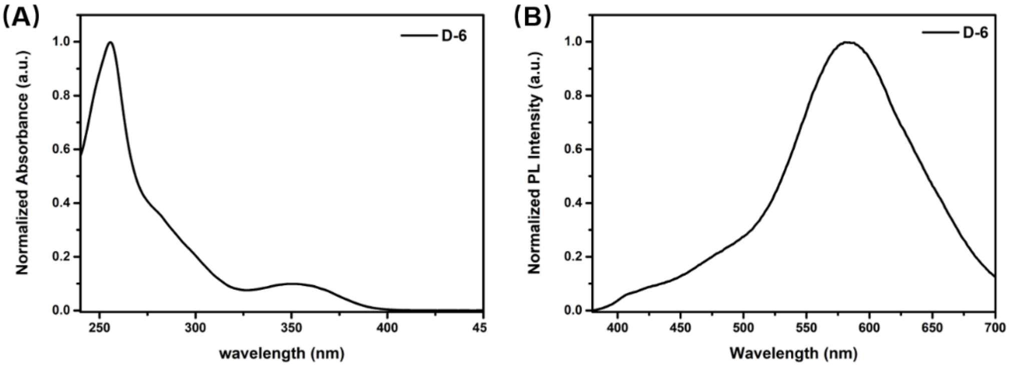

Figure S1 (A) The absorption spectra of **D-6** in Dichloromethane at a concentration of $1x10^{-5}$M. (B)The fluorescence spectra of **D-6** in Dichloromethane at a concentration of $1x10^{-5}$M.

9. Job's Plot curves of D-6 and various ammonium salts

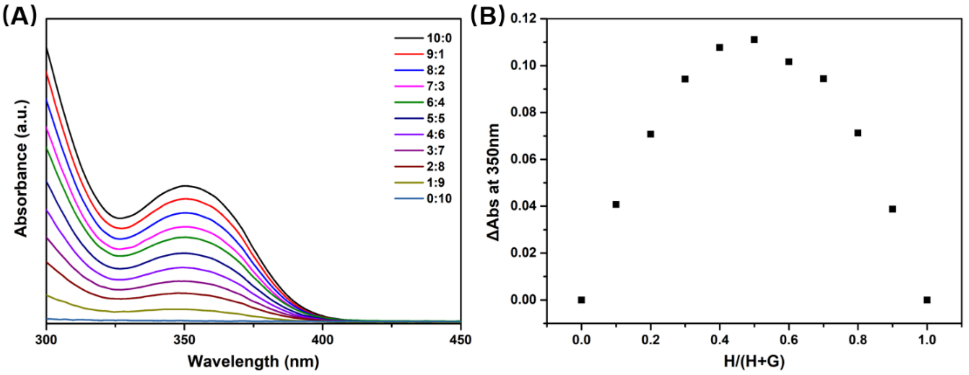

Figure S2. (A) A solution of **G1** in dichloromethane (5.0 × 10^{-5} M) and a solutions of **D-6** in dichloromethane (5.0 × 10^{-5} M) were mixed in a different ratio to prepare 11 samples. The UV/Vis absorption was measured for each samples, and the differences in the absorption at 350 nm were monitored. (B) The Job's plot of **D-6** and **G1** in dichloromethane at room temperature.

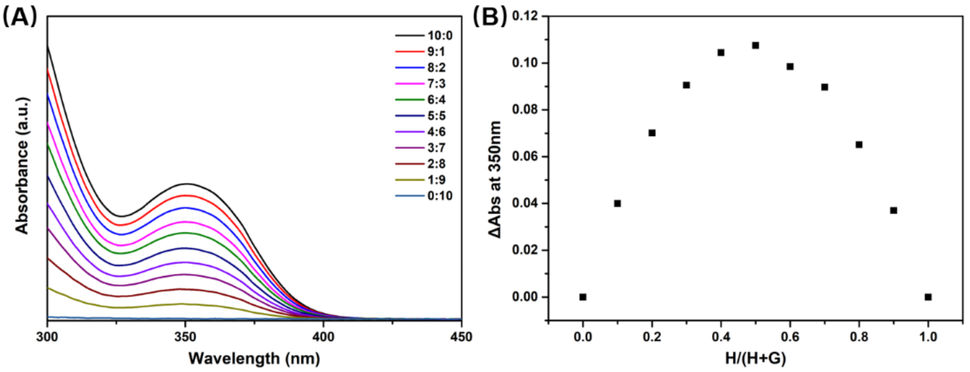

Figure S3. (A) A solution of **G2** in dichloromethane (5.0 × 10^{-5} M) and a solutions of **D-6** in dichloromethane (5.0 × 10^{-5} M) were mixed in a different ratio to prepare 11 samples. The UV/Vis absorption was measured for each samples, and the differences in the absorption at 350 nm were monitored. (B) The Job's plot of **D-6** and **G2** in dichloromethane at room temperature.

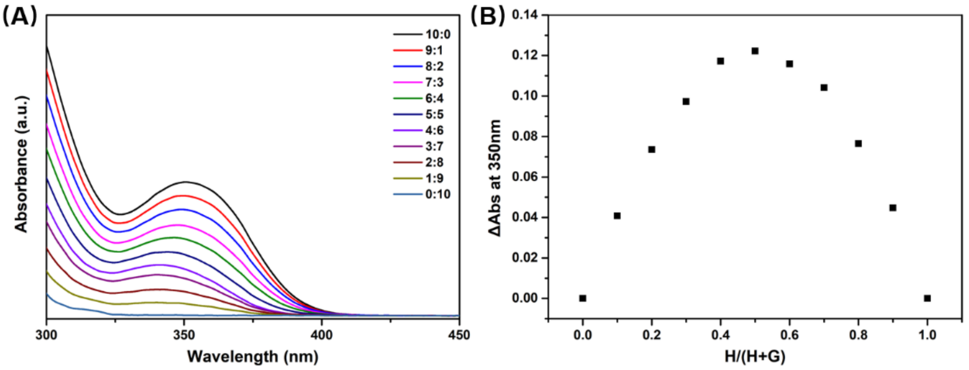

Figure S4. (A) A solution of **G3** in dichloromethane (5.0 × 10^{-5} M) and a solutions of

D-6 in dichloromethane (5.0 × 10^{-5} M) were mixed in a different ratio to prepare 11 samples. The UV/Vis absorption was measured for each samples, and the differences in the absorption at 350 nm were monitored. (B) The Job's plot of **D-6** and **G3** in dichloromethane at room temperature.

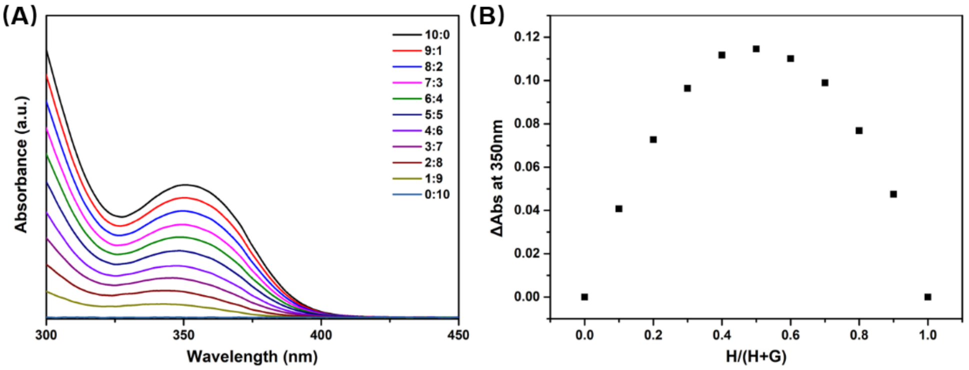

Figure S5. (A) A solution of **G4** in dichloromethane (5.0 × 10^{-5} M) and a solutions of **D-6** in dichloromethane (5.0 × 10^{-5} M) were mixed in a different ratio to prepare 11 samples. The UV/Vis absorption was measured for each samples, and the differences in the absorption at 350 nm were monitored. (B) The Job's plot of **D-6** and **G4** in dichloromethane at room temperature.

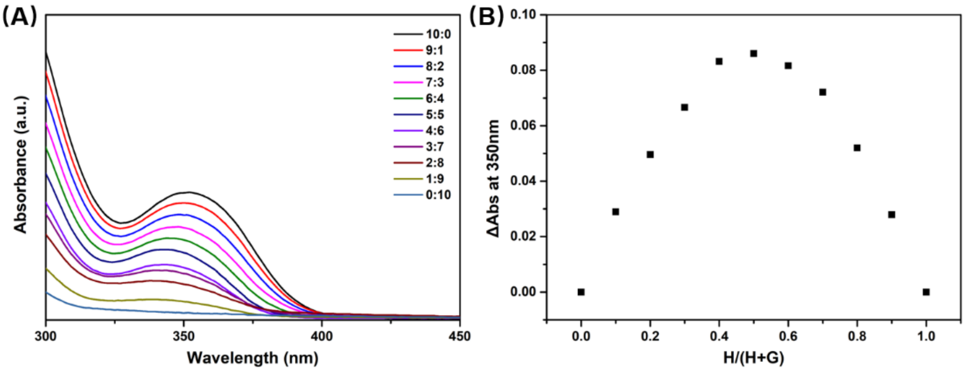

Figure S6. (A) A solution of **G5** in dichloromethane (5.0 × 10^{-5} M) and a solutions of **D-6** in dichloromethane (5.0 × 10^{-5} M) were mixed in a different ratio to prepare 11 samples. The UV/Vis absorption was measured for each samples, and the differences in the absorption at 350 nm were monitored. (B) The Job's plot of **D-6** and **G5** in dichloromethane at room temperature.

10. UV/Vis titration spectra of various ammonium salts for D-6. Determination of the association constant (Ka) between D-6 and Guests. http://app.supramolecular.org/bindfit/.

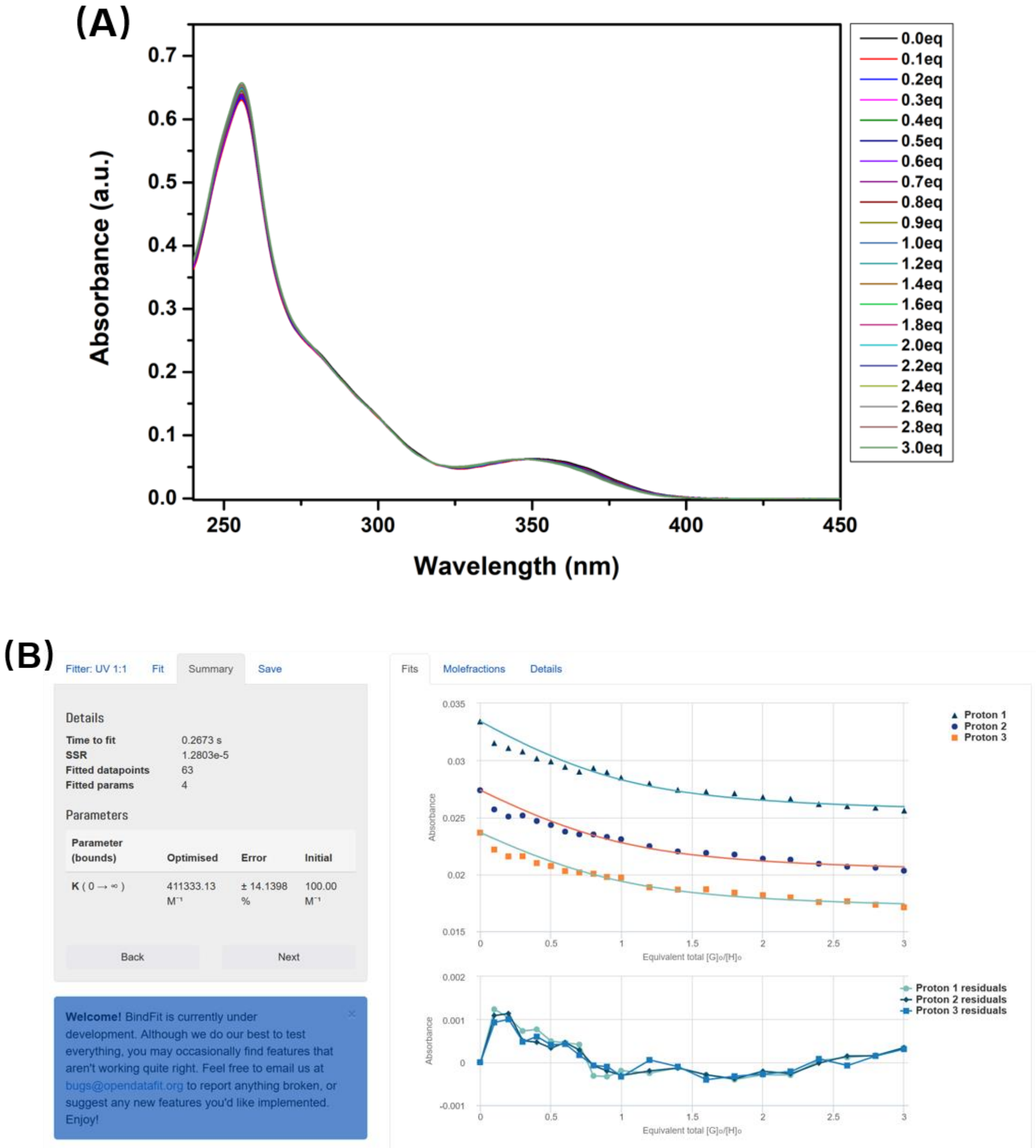

Figure S7 (A)UV–vis spectral titrations of **D-6** showing the spectral response produced upon the addition of **G1**. [**D-6**]: 10 μM; solvent: Dichloromethane. (B) The binding constant between **D-6** and **G1** is obtained from the website by UV titration data.

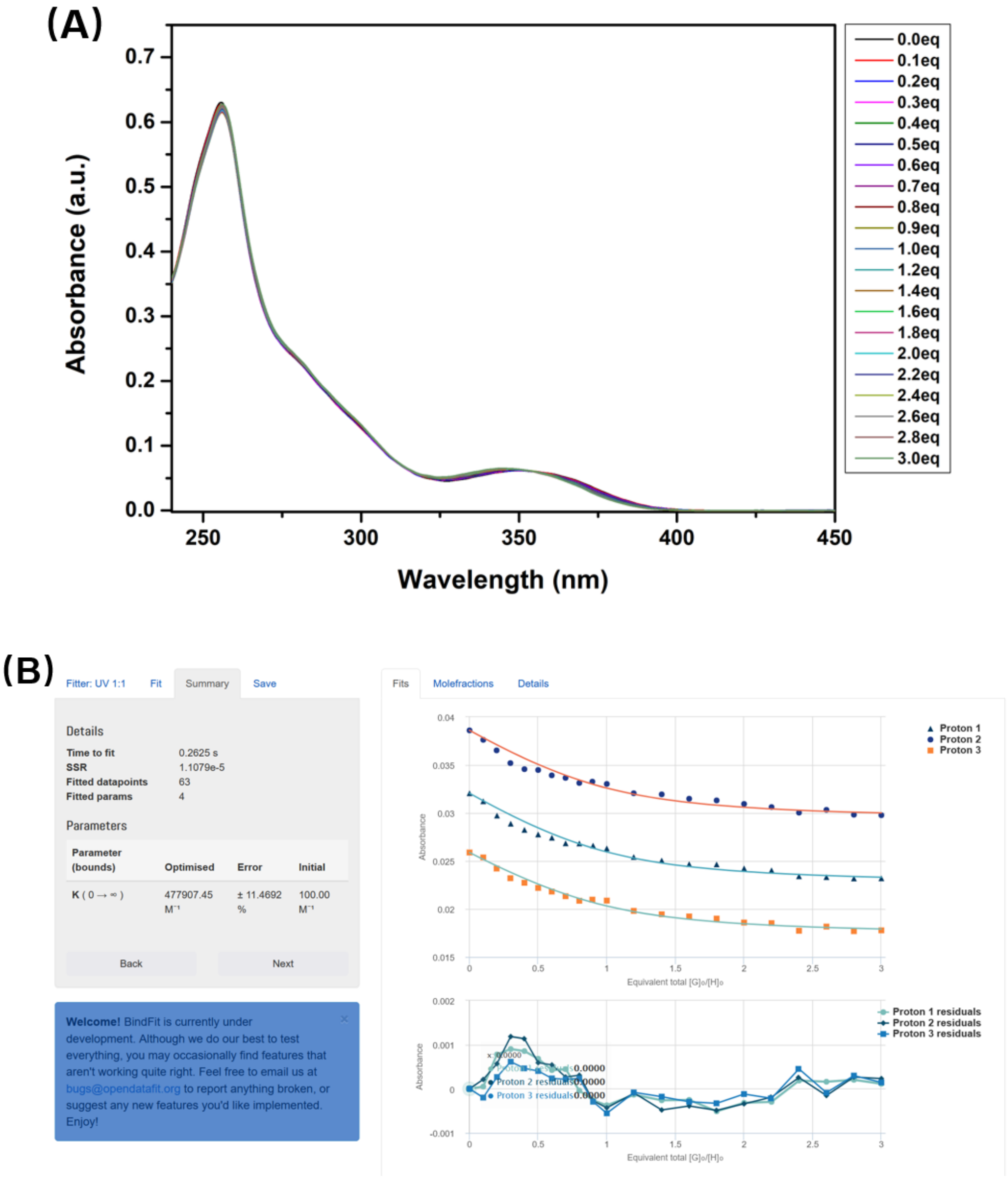

Figure S8 (A)UV–vis spectral titrations of **D-6** showing the spectral response produced upon the addition of **G2**. [**D-6**]: 10 μM; solvent: Dichloromethane. (B) The binding constant between **D-6** and **G2** is obtained from the website by UV titration data.

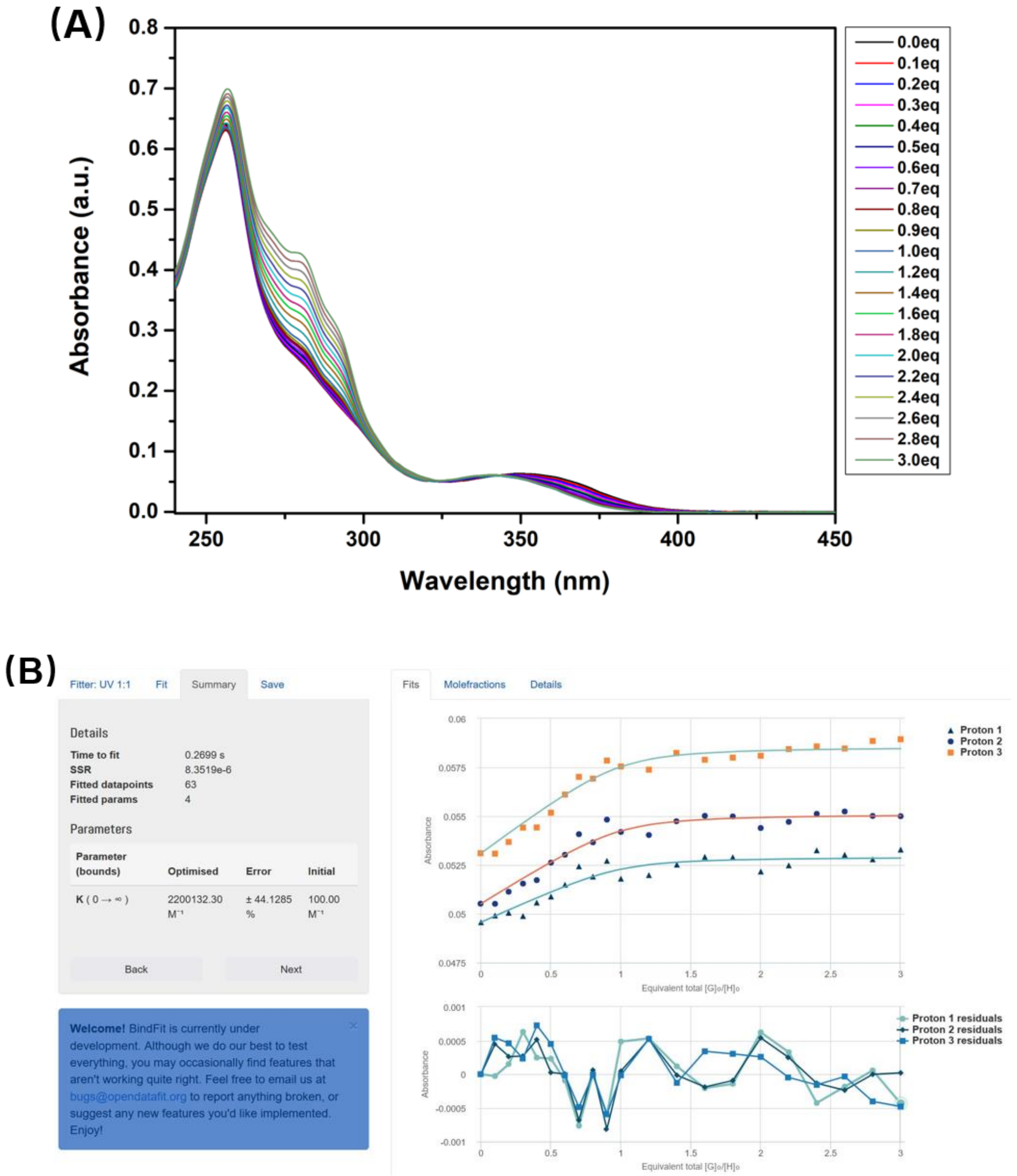

Figure S9 (A)UV–vis spectral titrations of **D-6** showing the spectral response produced upon the addition of **G3**. [**D-6**]: 10 μM; solvent: Dichloromethane. (B) The binding constant between **D-6** and **G3** is obtained from the website by UV titration data.

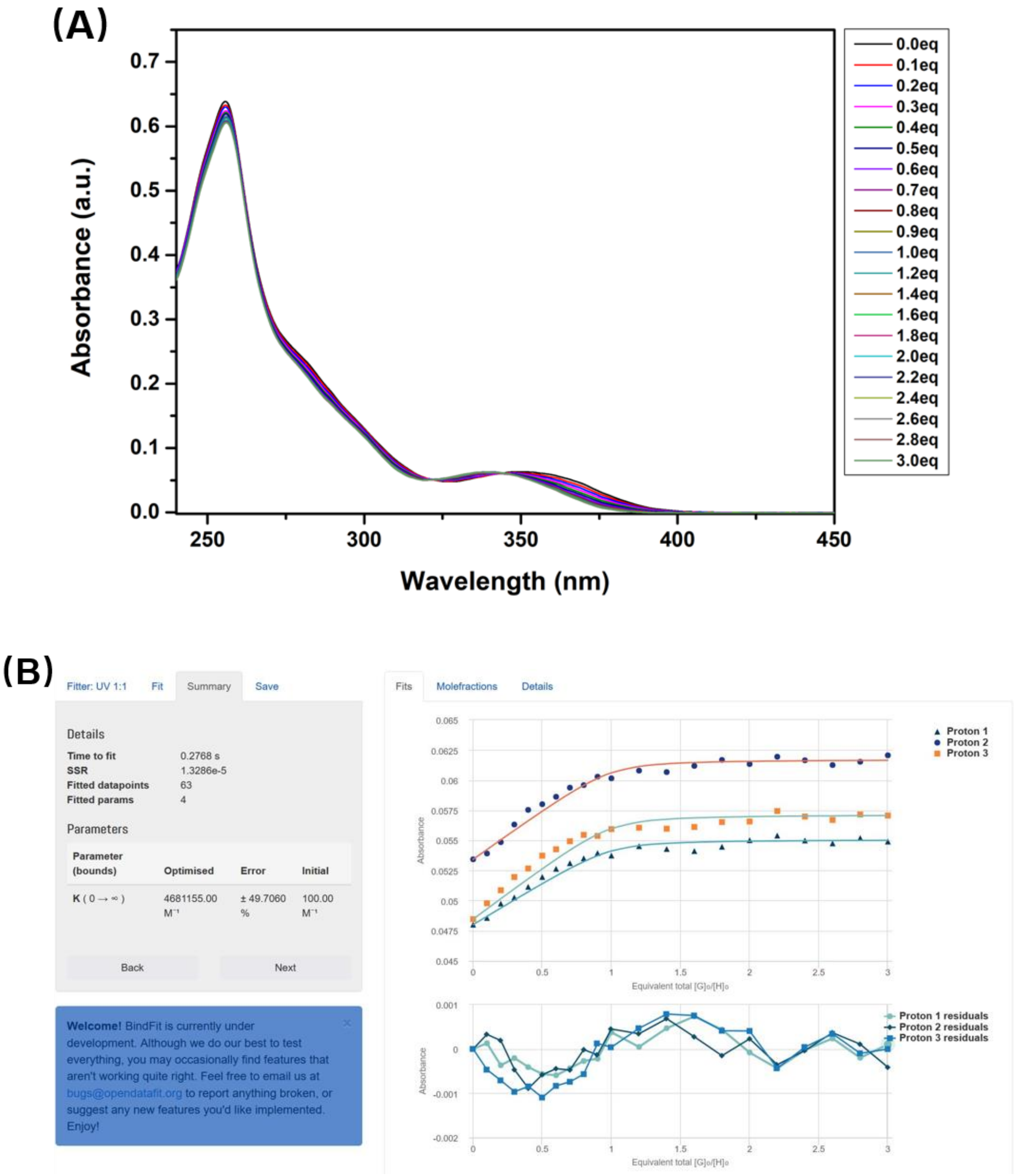

Figure S10 (A)UV–vis spectral titrations of **D-6** showing the spectral response produced upon the addition of **G4**. [**D-6**]: 10 μM; solvent: Dichloromethane. (B) The binding constant between **D-6** and **G4** is obtained from the website by UV titration data.

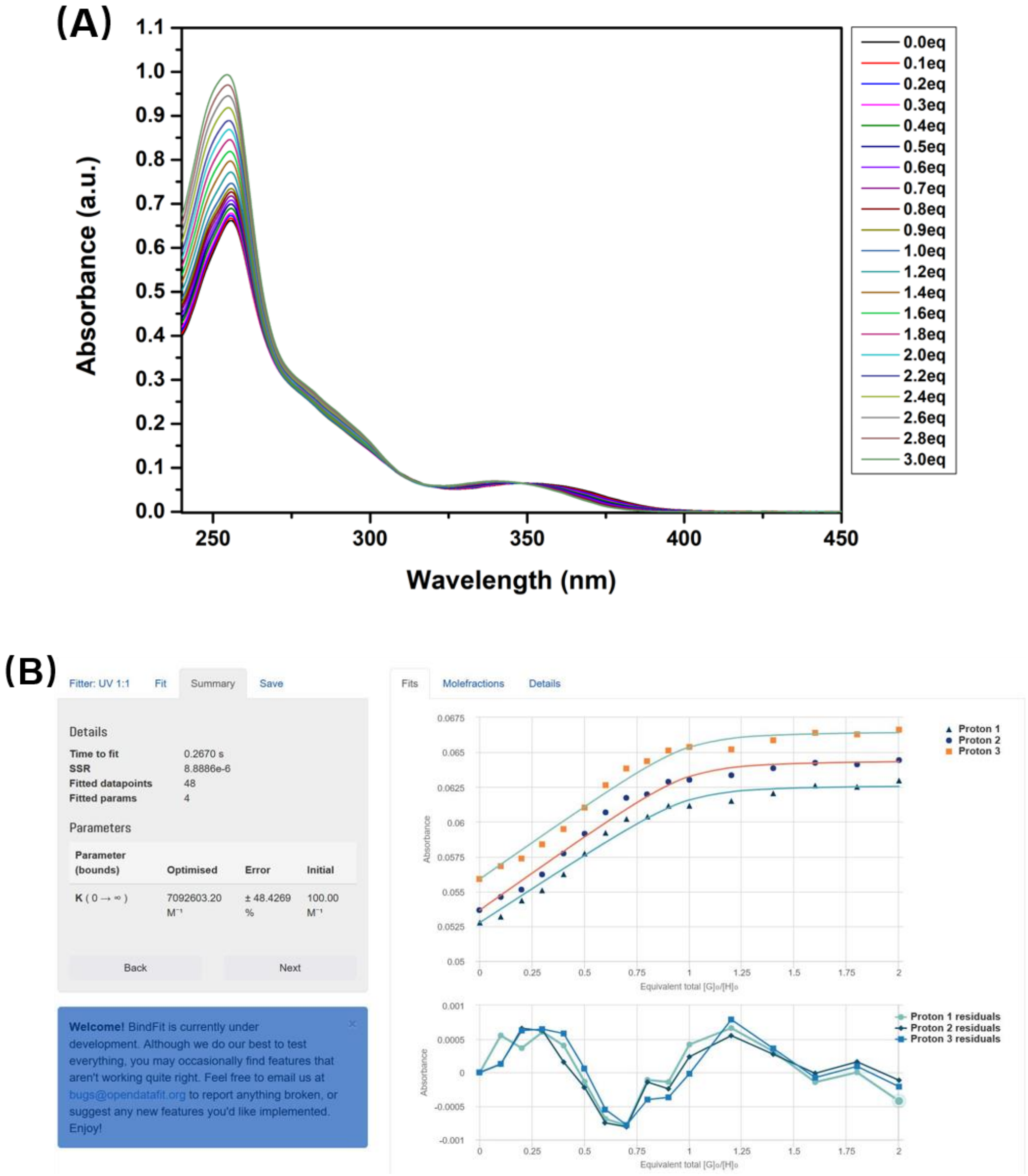

Figure S11 (A)UV–vis spectral titrations of **D-6** showing the spectral response produced upon the addition of **G5**. [**D-6**]: 10 μM; solvent: Dichloromethane. (B) The binding constant between **D-6** and **G5** is obtained from the website by UV titration data.

11. Optical properties and fluorescence spectra of various ammonium salts for D-6

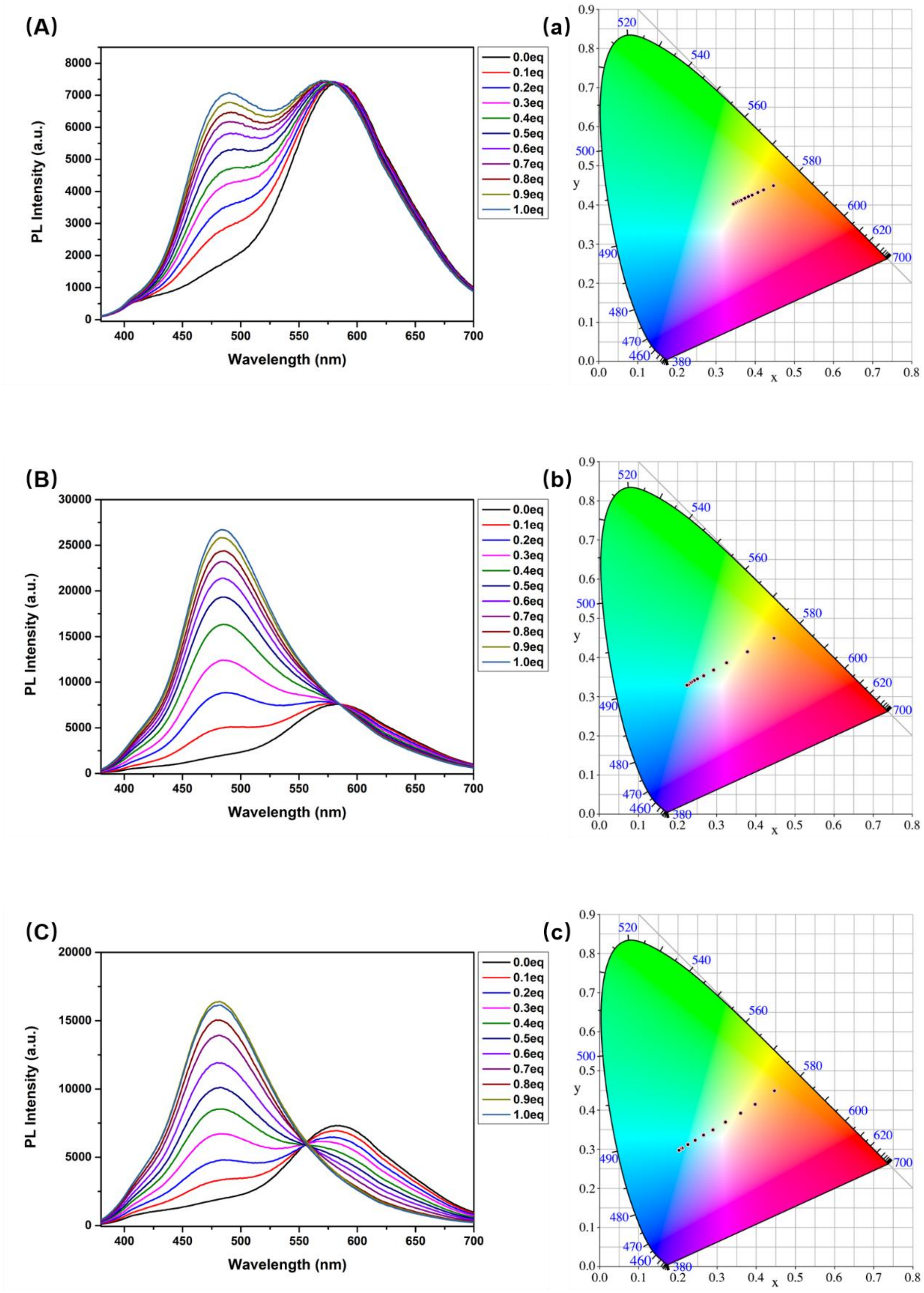
(A)
(B)
(C)
(a)
(b)
(c)
PL Intensity (a.u.)
Wavelength (nm)
0.0eq
0.1eq
0.2eq
0.3eq
0.4eq
0.5eq
0.6eq
0.7eq
0.8eq
0.9eq
1.0eq
x
y
520
540
560
580
600
620
700
500
490
480
470
460
380

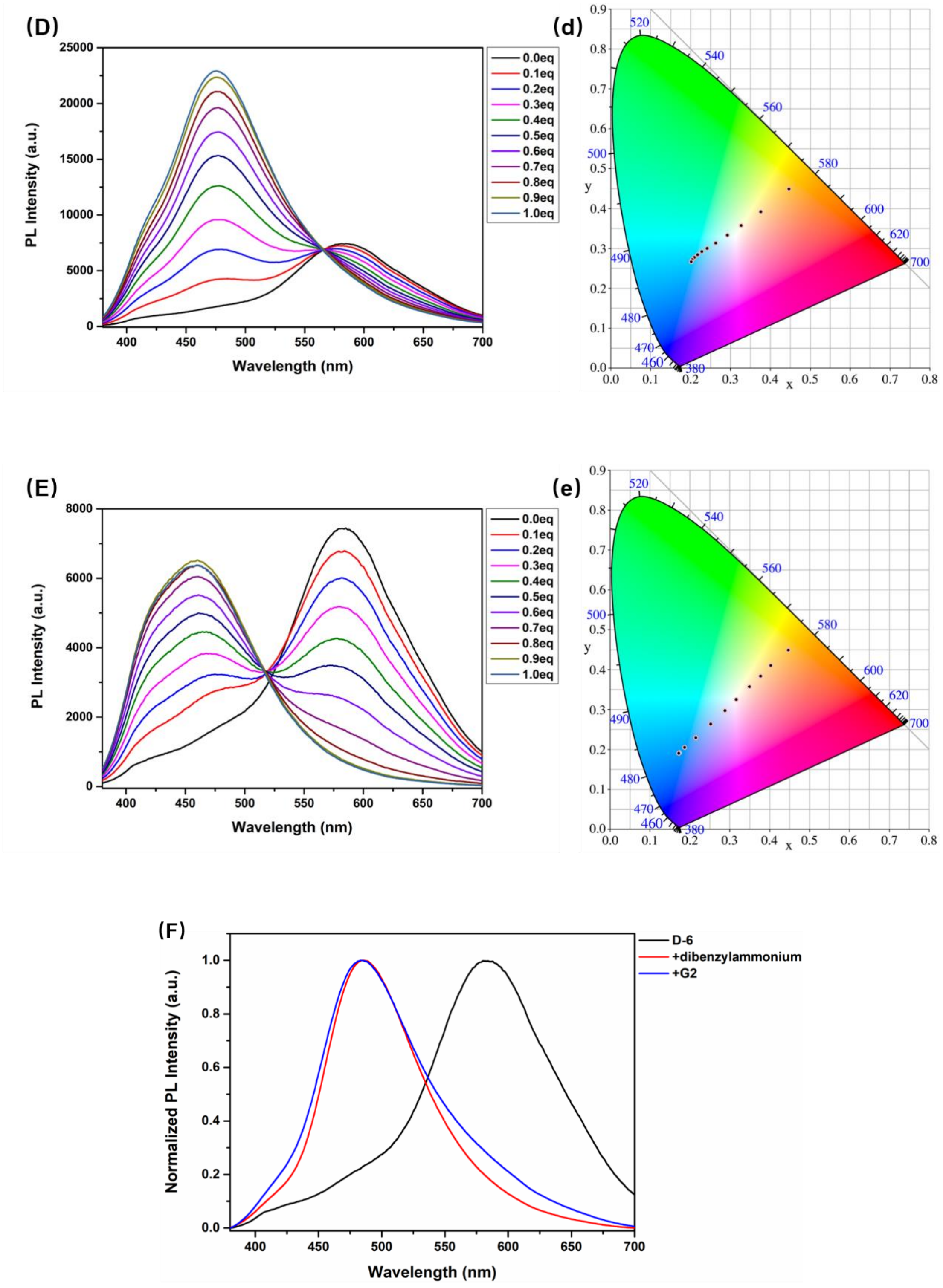

Figure S12 Fluorescence spectral titrations of **D-6** showing the spectral response produced upon the addition of (A) **G1**, (B) **G2**, (C) **G3**, (D) **G4** (E) **G5**. λ_{ex}: 360 nm [**D-6**]: 10 μM; solvent: Dichloromethane. Chromaticity coordinates (CIE) for **D-6** recorded in the presence of varying quantities of (a) **G1**, (b) **G2**, (c) **G3**, (d) **G4**, (e) **G5** (from 0 to 1.0 equiv). (F) Fluorescence emission after the addition of dibenzylammonium and G2 to D-6, respectively.

Table S1 Quantum yields and fluorescence lifetimes of the host-guest complexes of D-6 and different guests

	QY	λ_{probe} (nm)	τ	
D-6	3.4%	580nm	τ_1=8.24 ns (100%)	
D-6+G1	5.2%	480nm	τ_1=2.02 ns (11%)	τ_2=10.84 ns (89%)
D-6+G2	11.8%	480nm	τ_1=2.34 ns (7.9%)	τ_2=10.76 ns (92.1%)
D-6+G3	9.7%	480nm	τ_1=2.00 ns (11.3%)	τ_2=9.73 ns (88.7%)
D-6+G4	14.6%	480nm	τ_1=2.28 ns (9.4%)	τ_2=10 ns (90.6%)
D-6+G5	3.3%	450nm	τ_1=2.35 ns (24.6%)	τ_2=9.12 ns (75.4%)

The quantum yield was calculated using β-Carboline as the reference compound, and the excitation wavelength of λ_{ex}=360nm was chosen. Lifetime was measured by using a TCSPC system with a pulsed hydrogen-filled lamp as the excitation source (λ_{ex} = 375 nm).

12. ^{1}H NMR, ^{13}C NMR, and HRMS spectra

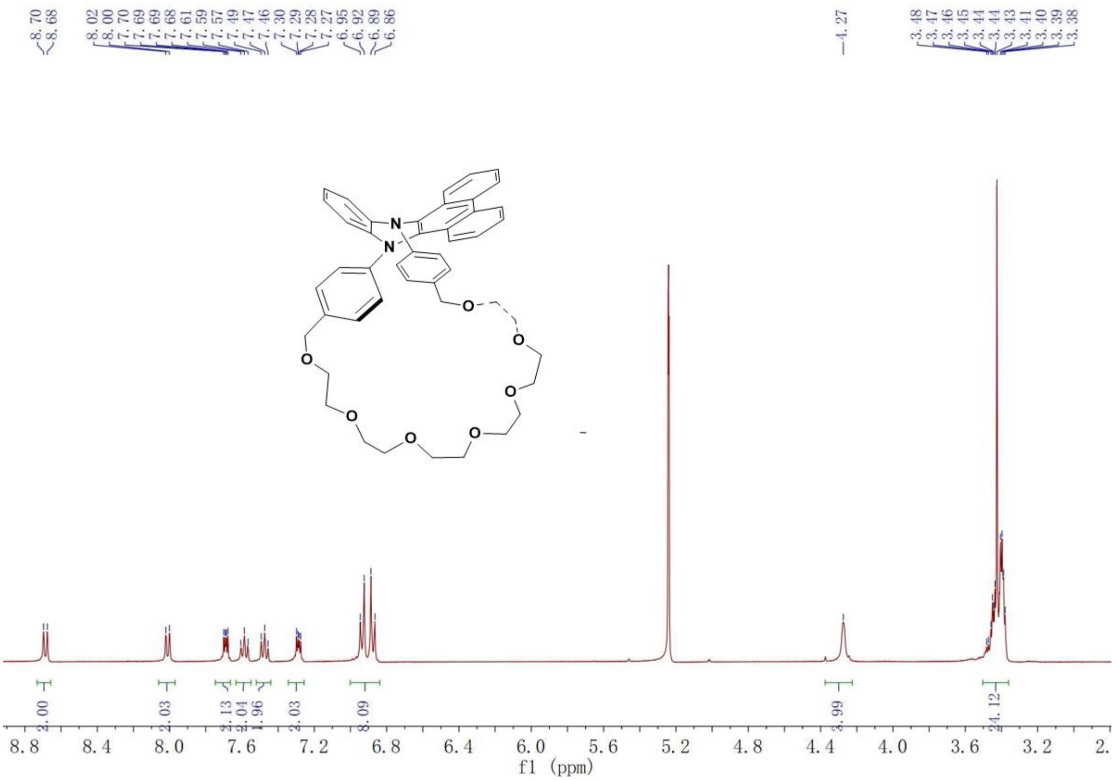

Figure S13 ^{1}H NMR (CD_2Cl_2, 400 MHz, 298K) spectrum of **D-6**

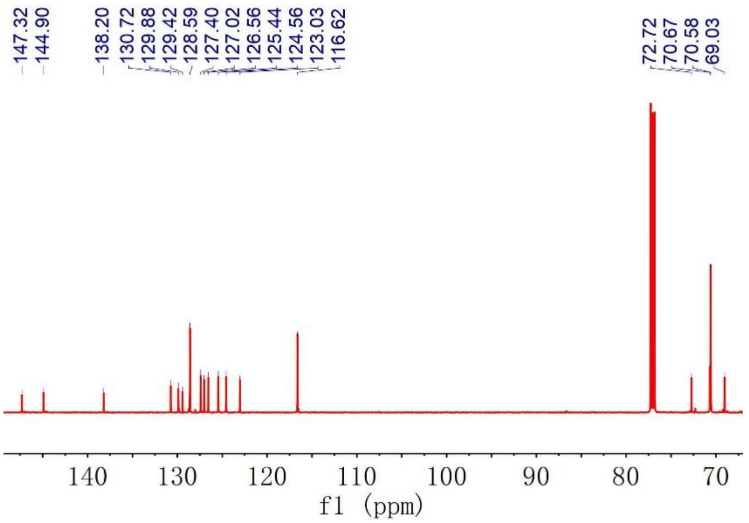

Figure S14 ^{13}C NMR ($CDCl_3$, 400 MHz, 298K) spectrum of compound **D-6**.

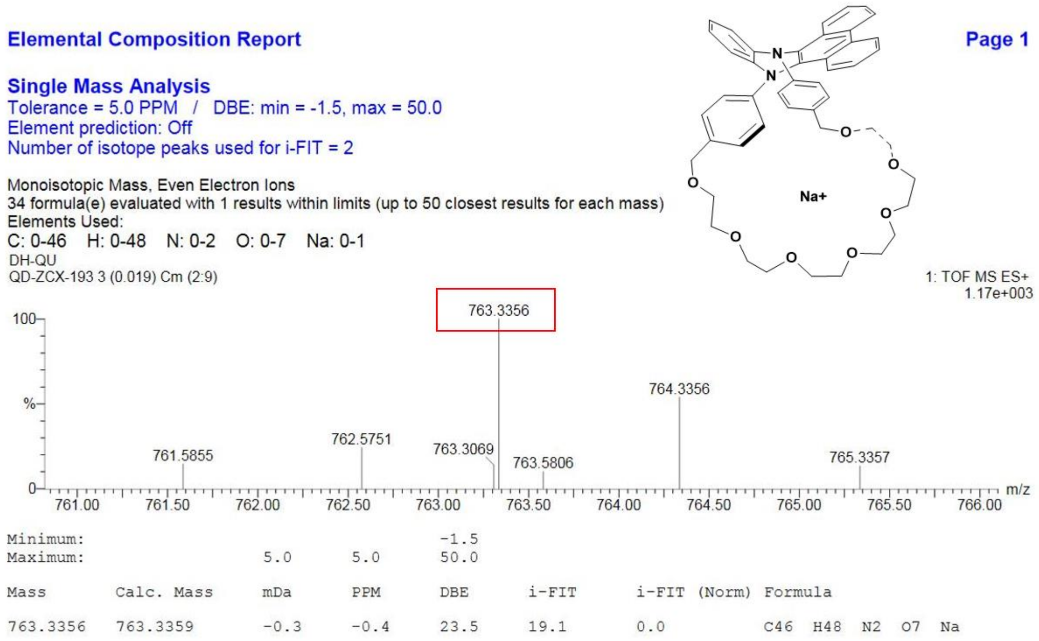

Figure S15 HRMS spectrum of compound **D-6**

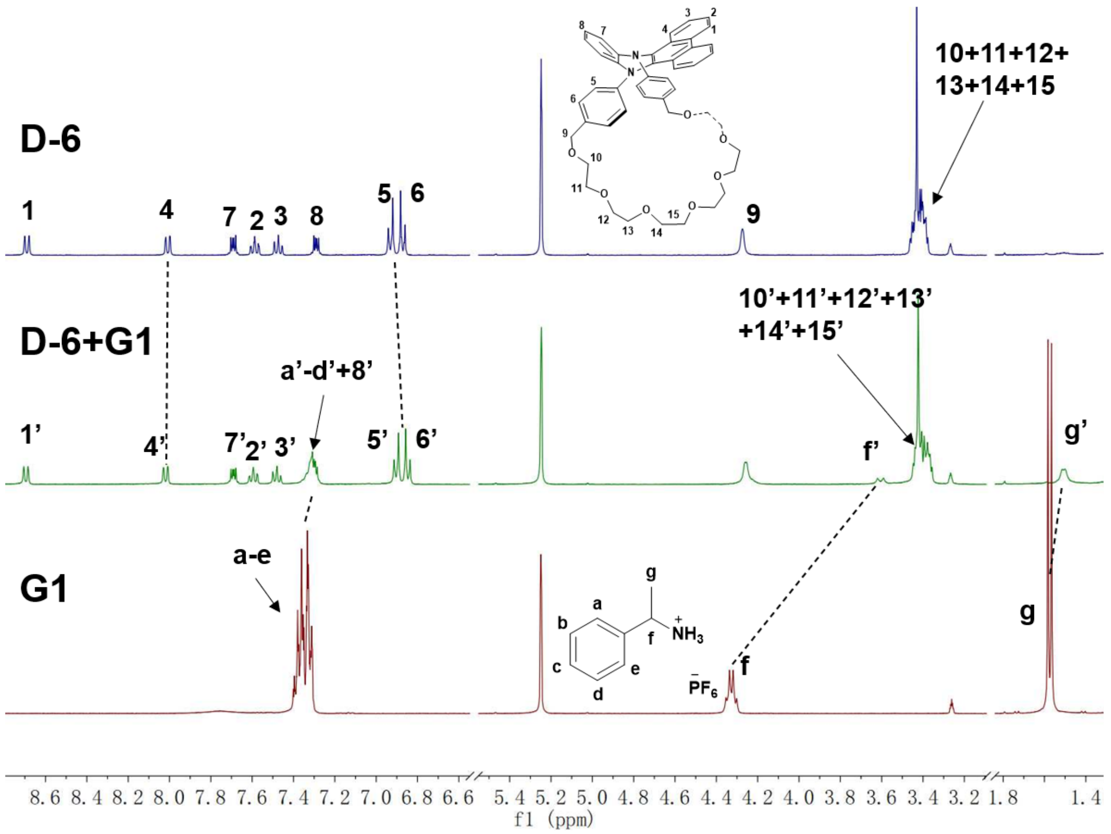

Figure S16 ^{1}H NMR (CD_2Cl_2:CD_3OD=95:5, 400 MHz, 298K) spectrum of **D-6**+**G1**

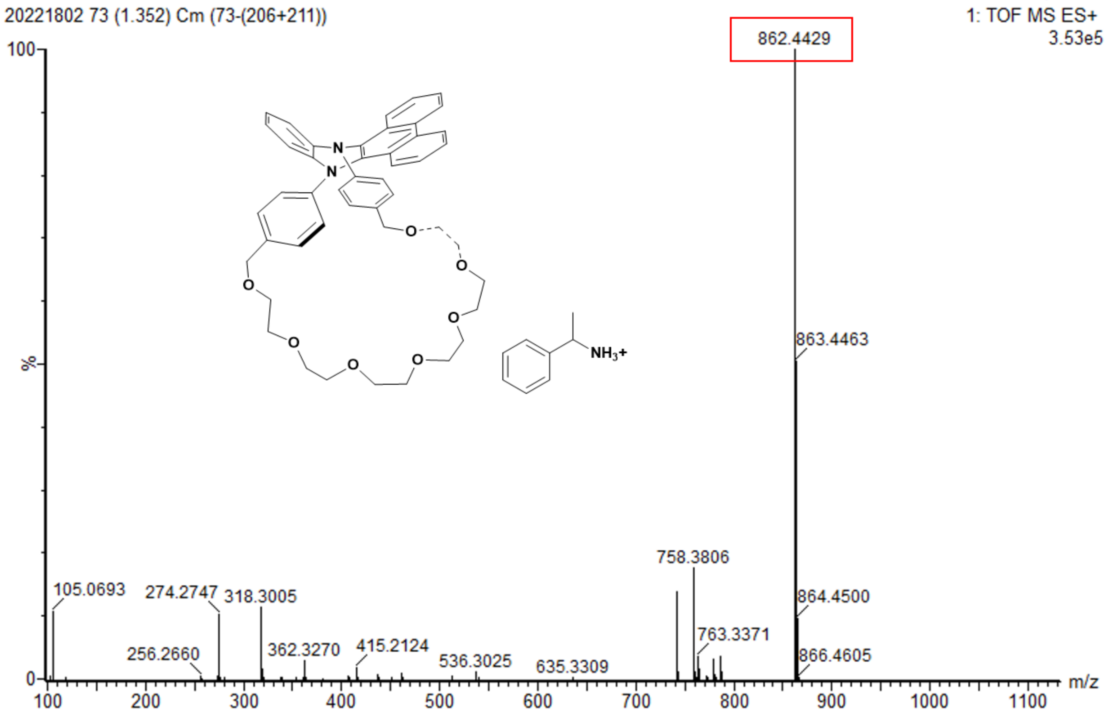

Figure S17 HRMS spectrum of compound **D-6**+**G1**

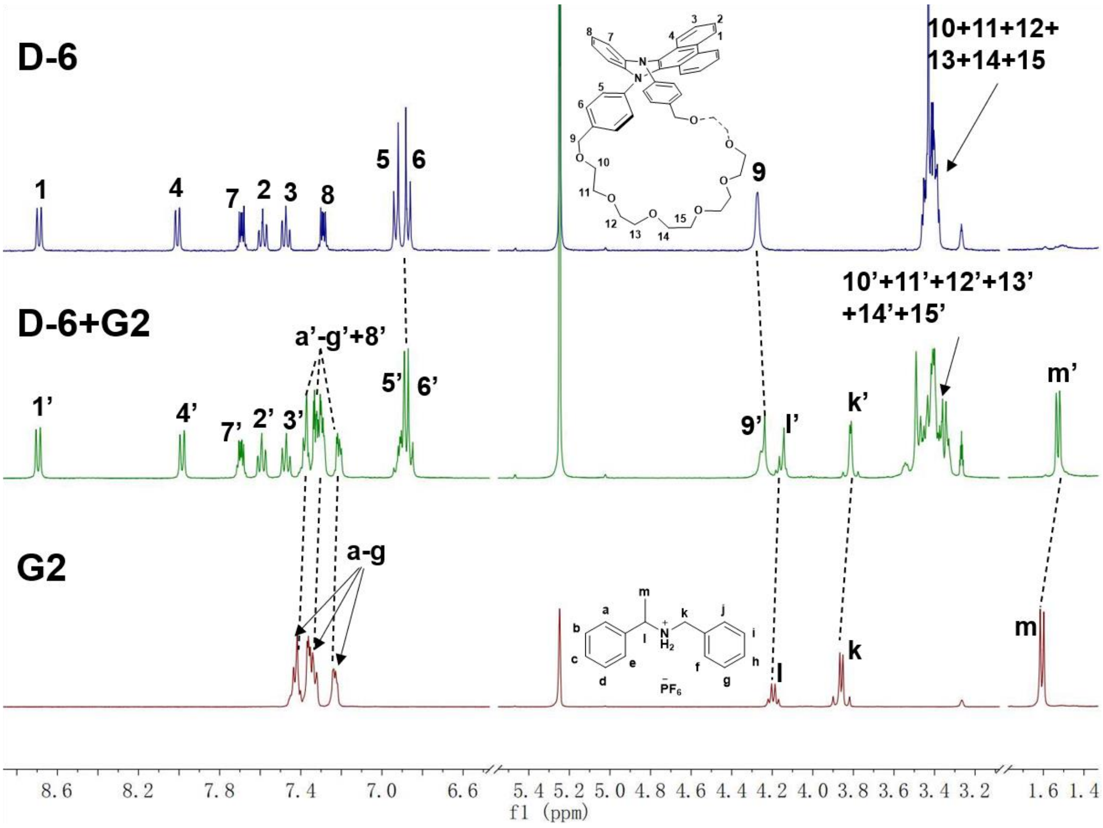

Figure S18 ^{1}H NMR (CD_2Cl_2:CD_3OD=95:5, 400 MHz, 298K) spectrum of **D-6**+**G2**

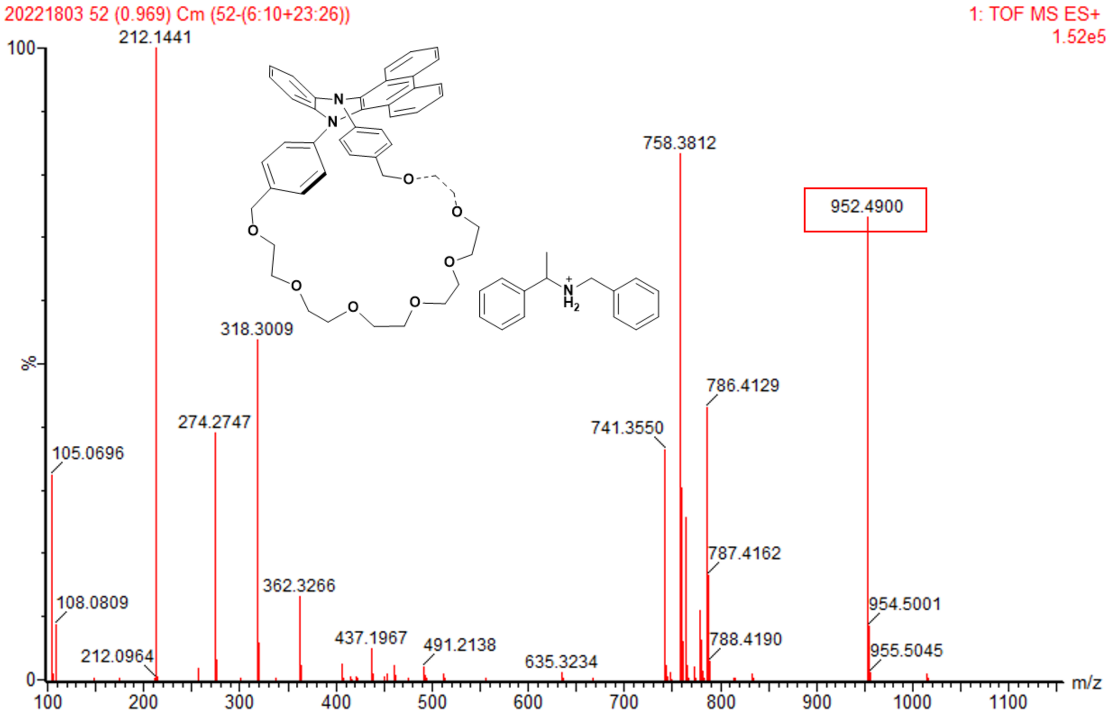

Figure S19 HRMS spectrum of compound **D-6**+**G2**

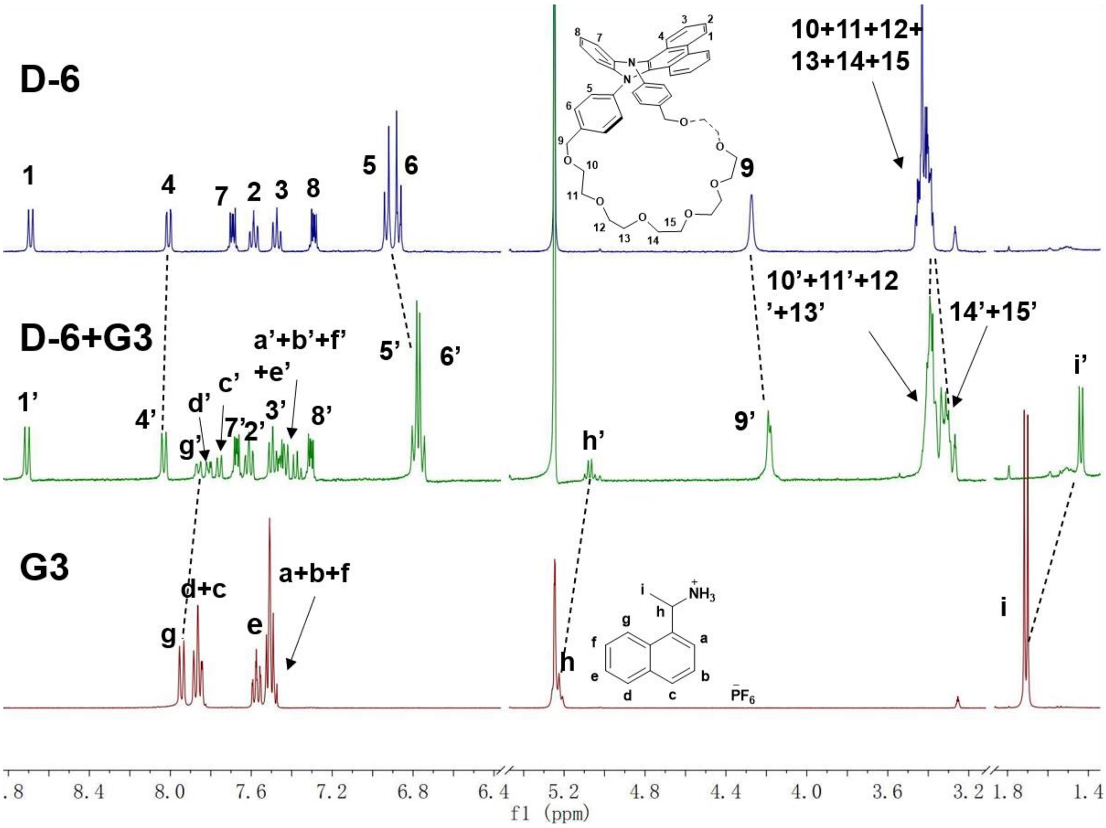

Figure S20 ^{1}H NMR (CD_2Cl_2:CD_3OD=95:5, 400 MHz, 298K) spectrum of **D-6**+**G3**

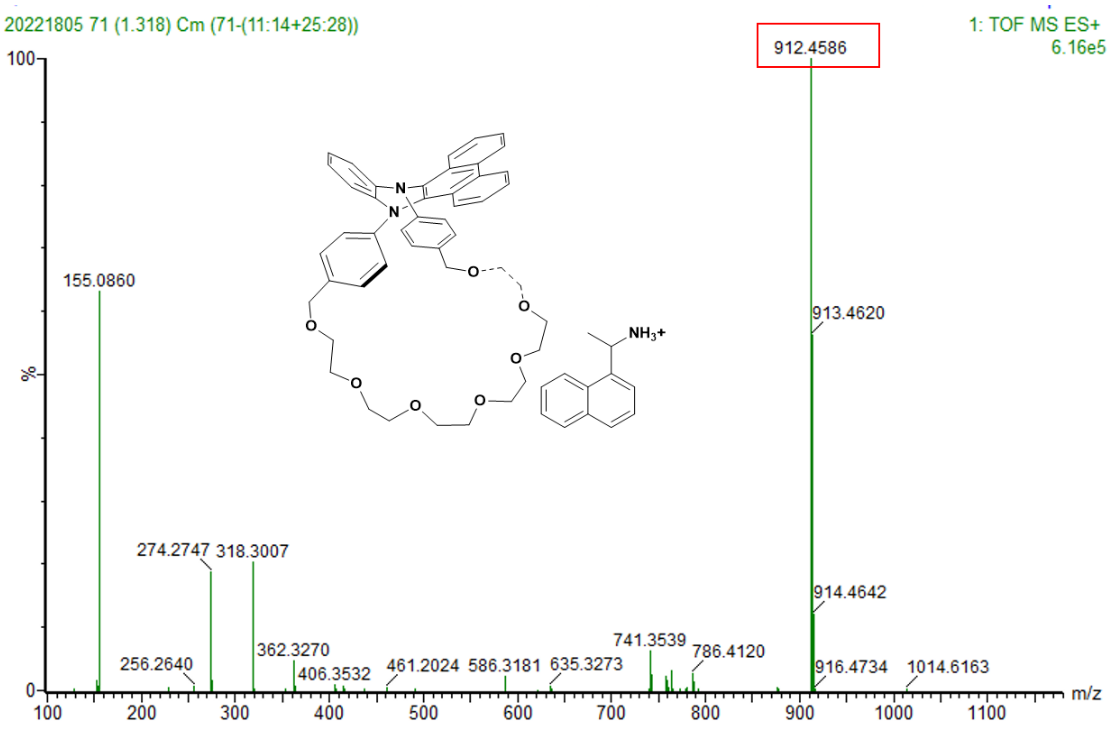

Figure S21 HRMS spectrum of compound **D-6**+**G3**

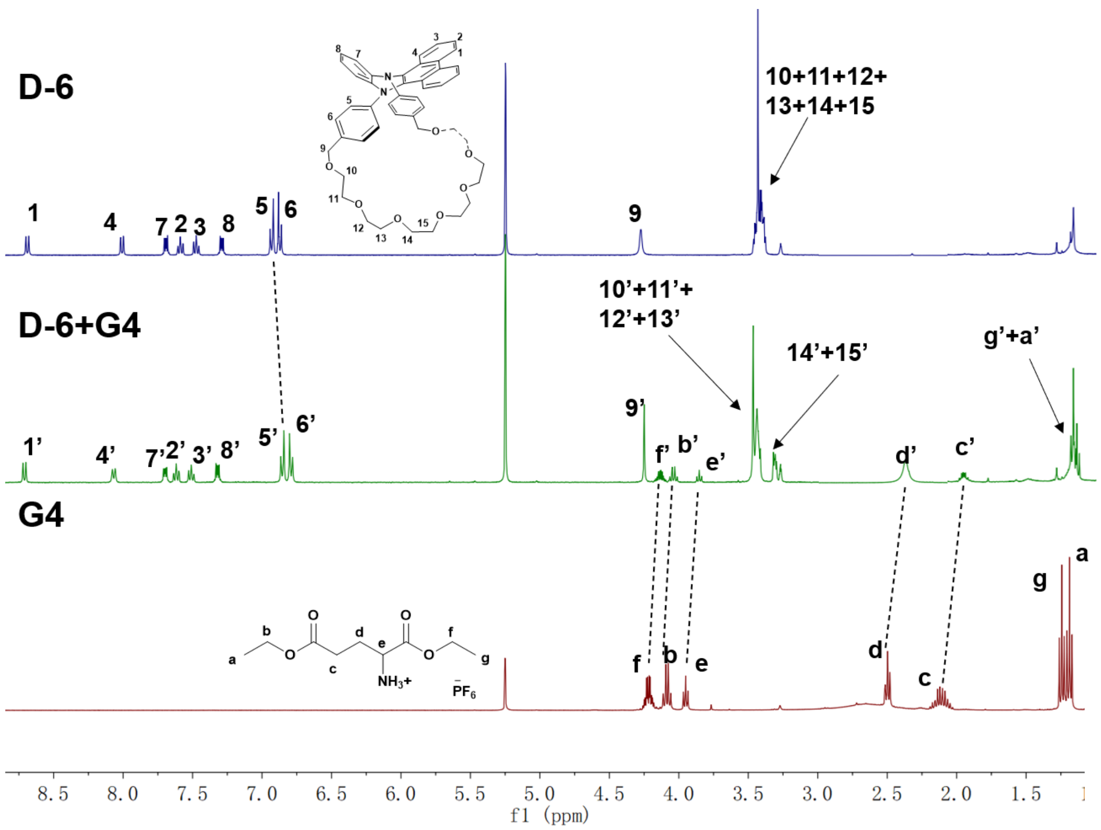

Figure S22 ^{1}H NMR (CD_2Cl_2:CD_3OD=95:5, 400 MHz, 298K) spectrum of **D-6**+**G4**

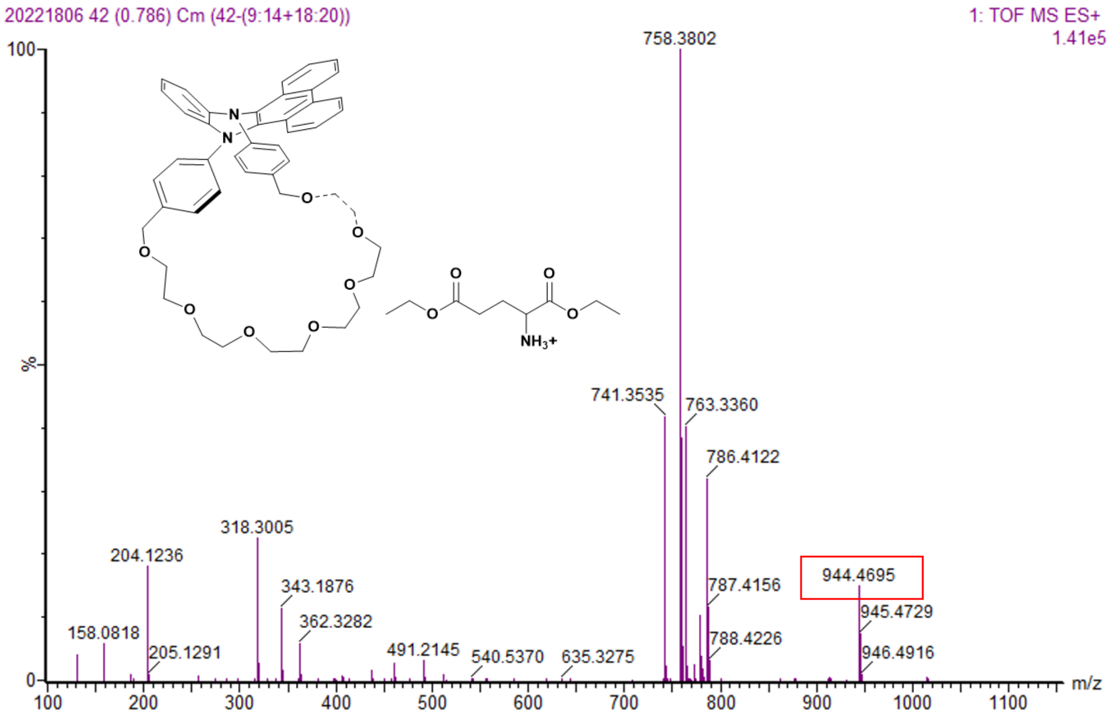

Figure S23 HRMS spectrum of compound **D-6**+**G4**

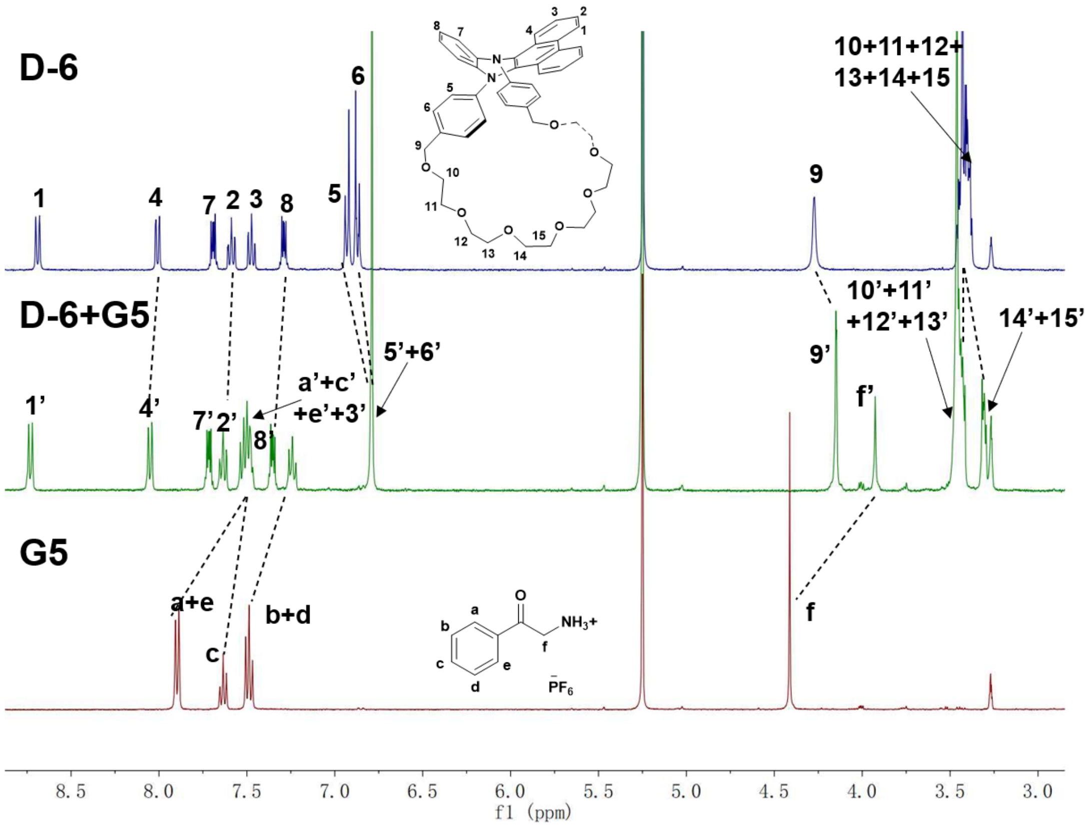

Figure S24 ^{1}H NMR (CD_2Cl_2:CD_3OD=95:5, 400 MHz, 298K) spectrum of **D-6**+**G5**

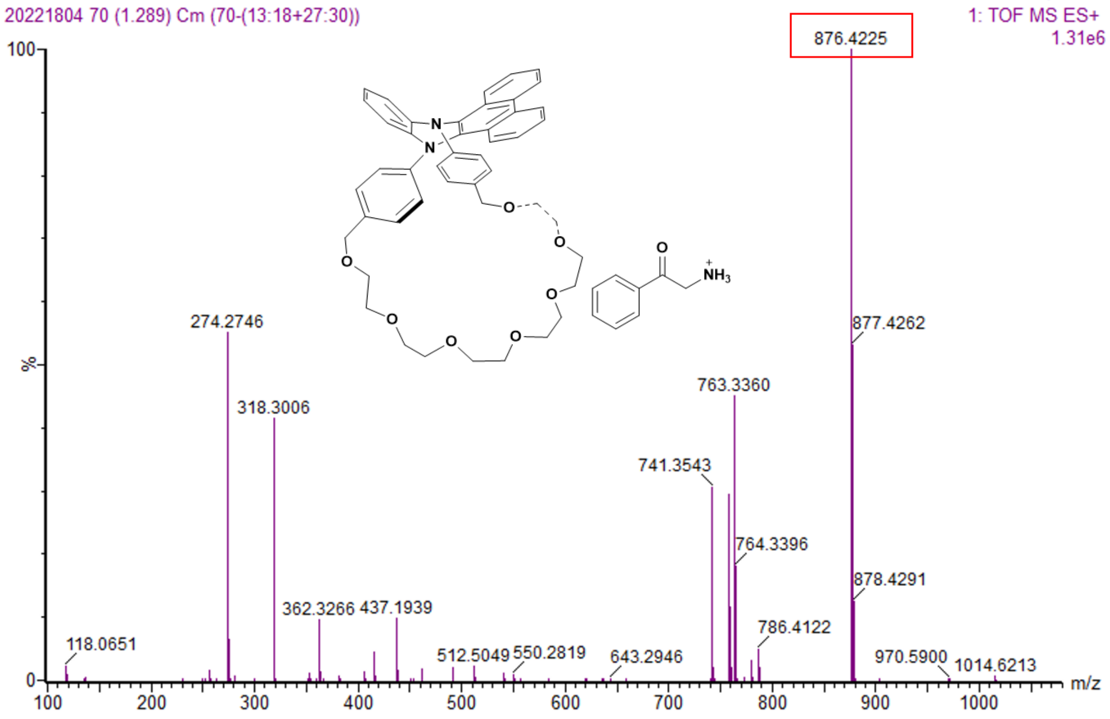

Figure S25 HRMS spectrum of compound **D-6**+**G5**
